# Supplementary material for: Epigenome-wide analysis of DNA methylation and coronary heart disease: a nested case-control study
Source: eLife. 2021 Sep 13;10:e68671. doi: 10.7554/eLife.68671 (PMC8585480; doi:10.7554/eLife.68671)
Supplement: Table 4—source data 6. [file elife-68671-table4-data6.docx]

**Table 4–Source Data 6. Association between quartile methylation level of identified CpGs and random glucose (mmol/L).**

|  | Quartile 2 | | Quartile 3 | | Quartile 4 | | P for trend  (adjusted for batch) | P for trend (adjusted for SVs) |  | Mediation effect | |
| --- | --- | --- | --- | --- | --- | --- | --- | --- | --- | --- | --- |
| CpGs | Effect size (se) | P | Effect size | P | Effect size | P |  |  |  | Proportion mediated, % | P |
| cg10400937 | -0.466 (0.225) | 0.038 | -0.015 (0.228) | 0.949 | 0.551 (0.231) | 0.017 | 0.003 | 0.013 |  | 6.72 | 0.107 |
| cg01545454 | -0.173 (0.229) | 0.449 | -0.163 (0.234) | 0.487 | 0.203 (0.234) | 0.385 | 0.006 | 0.111 |  | 9.58 | 0.097 |
| cg11754670 | -0.020 (0.230) | 0.931 | 0.316 (0.233) | 0.177 | 0.466 (0.236) | 0.049 | 0.005 | 0.652 |  | 36.39 | 0.086 |
| cg26334131 | -0.087 (0.233) | 0.708 | -0.398 (0.231) | 0.085 | -0.517 (0.234) | 0.028 | 0.018 | 0.234 |  | 32.70 | 0.109 |
| cg07219103 | 0.197 (0.229) | 0.391 | 0.462 (0.235) | 0.049 | 0.578 (0.244) | 0.018 | 0.032 | 0.856 |  | 6.17 | 0.135 |
| cg20302171 | -0.173 (0.229) | 0.450 | -0.128 (0.229) | 0.576 | -0.556 (0.234) | 0.018 | 0.027 | 0.692 |  | 15.77 | 0.123 |
| cg02591826 | 0.040 (0.229) | 0.861 | 0.286 (0.231) | 0.215 | 0.226 (0.236) | 0.339 | 0.080 | 0.509 |  | - | - |
| cg22794712 | 0.103 (0.231) | 0.655 | -0.049 (0.235) | 0.834 | 0.442 (0.240) | 0.066 | 0.118 | 0.982 |  | - | - |
| cg08106661 | 0.021 (0.233) | 0.929 | 0.232 (0.248) | 0.349 | 0.490 (0.263) | 0.063 | 0.137 | 0.990 |  | - | - |
| cg07560408 | 0.001 (0.232) | 0.998 | -0.168 (0.231) | 0.467 | -0.336 (0.235) | 0.153 | 0.293 | 0.647 |  | - | - |
| cg05681643 | -0.019 (0.229) | 0.932 | 0.019 (0.230) | 0.934 | -0.212 (0.239) | 0.375 | 0.345 | 0.130 |  | - | - |
| cg19583211 | 0.029 (0.230) | 0.901 | -0.075 (0.232) | 0.748 | 0.212 (0.233) | 0.364 | 0.408 | 0.352 |  | - | - |
| cg15833447 | -0.331 (0.230) | 0.150 | -0.153 (0.234) | 0.514 | -0.281 (0.236) | 0.235 | 0.423 | 0.837 |  | - | - |
| cg24792179 | -0.309 (0.233) | 0.184 | -0.250 (0.234) | 0.284 | -0.176 (0.239) | 0.462 | 0.485 | 0.155 |  | - | - |
| cg05740632 | -0.073 (0.231) | 0.752 | 0.083 (0.234) | 0.724 | -0.239 (0.235) | 0.309 | 0.501 | 0.815 |  | - | - |
| cg08484100 | 0.383 (0.229) | 0.095 | 0.223 (0.233) | 0.338 | -0.105 (0.235) | 0.655 | 0.407 | 0.676 |  | - | - |
| cg21210537 | 0.131 (0.234) | 0.577 | 0.370 (0.242) | 0.126 | 0.111 (0.249) | 0.657 | 0.617 | 0.192 |  | - | - |
| cg13311494 | -0.329 (0.230) | 0.154 | -0.089 (0.229) | 0.699 | -0.044 (0.233) | 0.852 | 0.704 | 0.195 |  | - | - |
| cg20562821 | 0.412 (0.233) | 0.077 | 0.361 (0.240) | 0.133 | -0.076 (0.241) | 0.752 | 0.721 | 0.602 |  | - | - |
| cg06358566 | -0.016 (0.228) | 0.944 | 0.023 (0.230) | 0.920 | 0.020 (0.233) | 0.931 | 0.756 | 0.169 |  | - | - |
| cg16639138 | -0.125 (0.227) | 0.581 | -0.253 (0.228) | 0.268 | 0.069 (0.229) | 0.764 | 0.988 | 0.242 |  | - | - |
| cg11630610 | -0.024 (0.234) | 0.918 | -0.044 (0.230) | 0.850 | -0.014 (0.233) | 0.953 | 0.857 | 0.863 |  | - | - |
| cg10643850 | 0.004 (0.234) | 0.987 | -0.136 (0.233) | 0.559 | -0.175 (0.236) | 0.458 | 0.946 | 0.070 |  | - | - |
| cg02386575 | 0.102 (0.229) | 0.657 | 0.213 (0.228) | 0.352 | -0.008 (0.231) | 0.971 | 0.978 | 0.480 |  | - | - |
| cg23398826 | 0.154 (0.229) | 0.501 | 0.134 (0.231) | 0.563 | -0.119 (0.233) | 0.609 | 0.955 | 0.781 |  | - | - |

CpG = cytosine-phosphoguanine site; SE = standard error; SV = surrogate variable.

Effect size is for the comparison with quartile 1. Multivariable model was adjusted for: age, sex, education level, marital status, smoking, drinking, physical activity, diet score, body mass index, fasting time, study area, treatment of diabetes, and batch or SVs. Mediation analysis was performed for significant CpGs (P for trend < 0.05) only.
