# Supplementary material for: Epigenome-wide analysis of DNA methylation and coronary heart disease: a nested case-control study
Source: eLife. 2021 Sep 13;10:e68671. doi: 10.7554/eLife.68671 (PMC8585480; doi:10.7554/eLife.68671)
Supplement: Supplementary file 2. — (A) Number of methylation markers associated with incident coronary heart disease across the range of P thresholds in the epigenome-wide association study. (B) Manhattan plot (A) and QQ plot (B) of the P values of the associations between each cytosine-phosphoguanine (CpG) site and incident coronary heart disease. In the Manhattan plot, the red line represents -log10(P) at false discovery rate (FDR) = 0.05. (C) Gene enrichment analysis of 2106 probes from the brown module which was significantly associated with coronary heart disease. (D) Associations of 25 significant CpGs with risk of coronary heart disease among 880 participants without usage of blood pressure lowering drug. (E) Association between quartile methylation level of identified CpGs and systolic blood pressure* (mmHg). (F) Association between quartile methylation level of identified CpGs and diastolic blood pressure* (mmHg). (G) The annotated or nearest annotated gene of the identified CHD-associated CpGs in our study and the previous GWAS finding. [file elife-68671-supp2.docx]

**Supplementary file 2A. Number of methylation markers associated with incident coronary heart disease across the range of P thresholds in the epigenome-wide association study.**

| P threshold | Number of markers | |
| --- | --- | --- |
|  | Observed | Expected^*^ |
| P < 10^-7^ | 2 | 0.07 |
| P < 10^-6^ | 3 | 0.75 |
| P < 10^-5^ | 22 | 7.5 |
| P < 10^-4^ | 180 | 75 |
| P < 10^-3^ | 1,245 | 748 |
| P < 0.01 | 10,106 | 7,477 |
| P < 0.05 | 44,799 | 37,386 |

*Expected number of markers was estimated as the number of tests (747,726) × [P threshold]


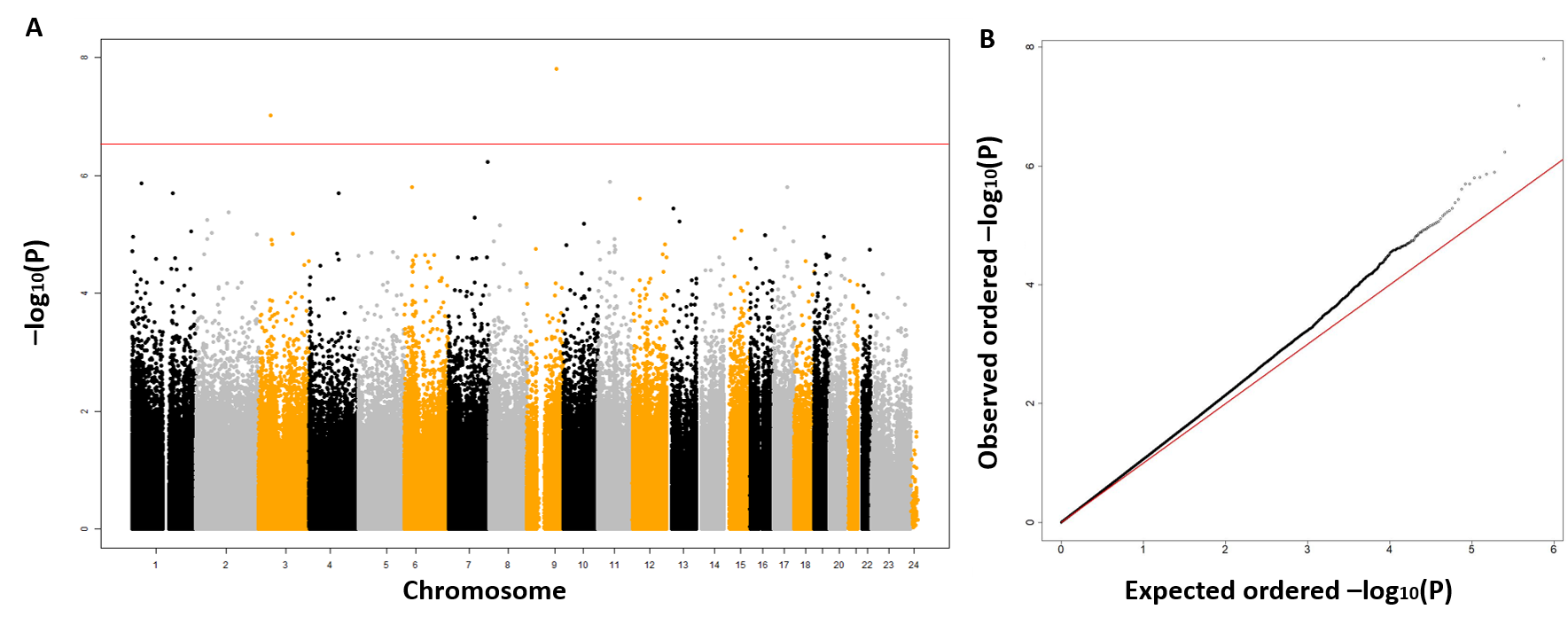
**Supplementary file 2B. Manhattan plot (A) and QQ plot (B) of the P values of the associations between each cytosine-phosphoguanine (CpG) site and incident coronary heart disease.** In the Manhattan plot, the red line represents -log10(P) at false discovery rate (FDR)=0.05.

**Supplementary file 2C. Gene enrichment analysis of 2,106 probes from the brown module which was significantly associated with coronary heart disease.**

| Category | Term | Number of genes in term | % | P  value | Genes | Fold Enrichment | Bonferroni | Benjamini |
| --- | --- | --- | --- | --- | --- | --- | --- | --- |
| GAD_DISEASE | Tobacco Use Disorder | 343 | 22.001 | 6.41E-11 | SCAF1, LTBP1, TPD52, SIDT2, PRKG1, VPS13D, ATXN10, ZFP90, CDCA2, RAPGEF4, ZNF106, CHRNA7, SMOX, RARB, CDH23, CEP295, OSBP2, BCR, POLE, TMEM132B, OLA1, PTPRT, ABCC12, WNK2, ICA1L, VTI1A, C1QB, RFC3, MYO18B, AQR, PRDM4, TRAPPC9, CEP350, HNF4A, CAMK4, PDE4DIP, FBXL7, KIF26B, PLCXD3, SLC2A10, MGAT5B, ENPP2, NFKBIA, CTIF, FAM19A5, NPHP4, ACE, DUSP16, PEMT, PPP2R2C, VWA3B, ABR, ST6GAL2, ZC3H18, AGBL4, TAOK3, TMEM120B, ANKMY1, ATP11A, HOMER1, ABCG8, NOTCH2, KCNJ6, FAM184A, GRK7, GOSR2, ZFHX3, ACAD10, RERE, PLCXD2, CDC42BPB, BTBD11, KCNH1, ELF1, PXDN, BACH2, JPH2, UVRAG, PDIA5, TTC28, MCF2L, VEPH1, WDR37, NOD2, PARN, MGRN1, RRP1B, DIP2C, SLC23A2, P4HA3, CHST11, EEF2K, SLC22A4, TICAM2, RBM47, ZFAT, DPP6, AGPAT4, AGPAT3, GOLGA3, FANCC, JAKMIP1, KCNMA1, SRPK2, TBX15, STX3, POLR1E, PIK3C2B, MICAL3, SDK1, CPXM2, HERC4, HERC3, RFTN1, ARHGEF10, FMN1, ARVCF, ATP6V1C2, NCK2, GRM3, STYK1, CDR2, PLXDC1, SIAH3, PLA2G6, BRDT, DENND3, PARD3, SLC39A11, TBC1D9, STAT4, NUMA1, SQSTM1, DGKD, NUP210, TEKT1, USP39, PEX14, BCL6, MYOM1, RPIA, TBC1D1, AGL, BCL9, ELMOD1, DLGAP2, ATRN, IPO11, MACROD2, DGKI, CAPN2, RASSF5, FBLN1, MEOX2, RPS6KA2, RAB35, CDON, WDR27, SH3RF3, ABCC3, CHN1, PHF21A, BIK, DPYD, CIT, APBB1, PHLDB2, DNM1, GNA13, GNA14, MTSS1, STIL, PLXNA4, MAU2, GRIK3, IL19, ZNF827, TLR5, ILDR1, MCM10, SLC25A21, CIB4, ANK2, ATG5, ROBO1, TBC1D14, RABGEF1, CPA2, ITPK1, MTUS2, CIITA, NBAS, PTPRN2, SLC22A23, ARID1B, FOXN3, TBC1D22A, ELL2, RNF138, ERC2, ERC1, CAMTA1, BBS4, ARFGAP3, STX8, MAMDC2, GRB2, DSCAML1, COL28A1, RABGAP1L, TRRAP, GREM2, MEIS1, CSMD2, WT1, CSMD1, SESN3, DOCK1, PPL, AUTS2, SH3TC2, AXIN1, OBSCN, TAF4, VAV3, SMCHD1, EHMT1, SPTBN5, UST, MYO1E, SLC6A13, NR4A2, ARID3B, DNHD1, TEAD1, MED13L, LHPP, FOXP1, SREBF2, CACNA2D4, DNA2, SLC4A10, RNF157, HIVEP3, CACNA1E, SPTA1, PIP4K2A, CACNA1C, HDAC9, PLEKHA2, CACNA1B, ST7, CDK19, ACOX2, MAD1L1, NRG3, CD8B, PRKAG2, WWC1, CAD, KLHL29, MAN2B2, GSN, TMEM59, AOAH, NARS2, RTF1, PSMD1, ZNF407, TPO, PDE8B, PAK1, SHC3, LOXL1, SHC4, DLG1, ANKS1A, SSBP3, COL23A1, NRXN3, PRKCH, PCDH9, LDLRAD3, PPM1F, NPTXR, TBCA, ZMIZ1, CAPN13, HHAT, PDE9A, WNT11, LCOR, DCC, ABLIM2, GALNT2, ZNF767P, SBNO2, ADAMTS14, HS3ST4, FKBP5, PPP1R12B, MGMT, LMF1, GLIS1, CTNND2, COMT, PRDM16, IVNS1ABP, CDKAL1, CCDC57, PKD1L1, APLP2, IARS, COL17A1, SH3GLB2, TNR, MYCBP, NFATC2, SCN5A, TNPO3, ECT2L, DLST, ADARB2, SCRN1, RCAN1, PAPLN, PARK2, MAN1C1, COL5A2, MEMO1, ATXN7L2, CADPS, ATXN1, ADI1, TMEM43, KCNN3, KDM4B, CABIN1, ATP8A2, IRF2, JAK2, NFIC, SH3BP2 | 1.354015 | 2.02E-07 | 2.02E-07 |
| GAD_DISEASE | Apoplexy\|Cerebral Hemorrhage\|Cerebral Hemorrhages\|Intracranial Hemorrhages\|Stroke\|Subarachnoid Hemorrhage | 38 | 2.437 | 1.47E-10 | CAMTA1, PACS2, PXDN, ATL1, PPP1R12B, RABGAP1L, PRKG1, CXCL12, ITM2C, ACE, WDR37, ACOT7, DOCK1, DIP2C, PIGG, NUP210, CUL9, ITPK1, SS18L1, TBL3, TAF4, CCDC86, COL23A1, SPTBN5, LZTR1, COL13A1, PTPRN2, SDK1, NADSYN1, WNK2, MYH9, TP73, ARVCF, LAMA3, TRAPPC9, SBF1, TBCD, NCOR2 | 3.250877 | 4.63E-07 | 2.32E-07 |
| GAD_DISEASE | Kidney Diseases | 25 | 1.604 | 1.67E-06 | IL6, PXDN, TBL3, COL23A1, ATL1, PTPRN2, PRKAG2, NADSYN1, PTPRT, WNK2, MYH9, ARVCF, ACOT7, ACE, WDR37, LAMA3, ATG5, TRAPPC9, PIGG, CERS2, TBCD, POU2F2, LIPC, ITPK1, SS18L1 | 2.994229 | 0.005251 | 0.001753 |
| **Annotation Cluster 1** | **Enrichment Score: 6.901** |  |  |  |  |  |  |  |
| UP_KEYWORDS | Metal-binding | 365 | 23.412 | 1.05E-12 | ADCY3, MASP1, GBGT1, PNKD, FAM20B, THOP1, CIAPIN1, SHH, CDH22, PLOD3, CUL9, ZFP90, ZNF397, ZNF106, RARB, CDH24, CDH23, RPP21, CIZ1, POLE, ZNF790, OLA1, PITPNM2, PITPNM3, PRDM4, PIAS4, HNF4A, NME1, XXYLT1, UBR5, TRMT13, ZGLP1, ACAP2, ZNHIT1, FDX1L, ZZZ3, PELO, ZNF382, PLA2G2F, ITGA2B, SIVA1, TRIM64C, MGAT5B, ZNF132, GNAI2, NEK2, ENPP2, CYP51A1, ACP5, MYO9A, PXN, PPAT, ADAP1, NR1H2, THTPA, ARIH2, ACE, ZNF225, CDADC1, CASZ1, LHX5, RCHY1, DNMT3B, NTHL1, DHX57, NR1H3, KLF5, ZNF529, BMP1, ZC3H18, AGBL4, CREBBP, ZNF621, ANKMY1, ATP11A, SMYD2, AARSD1, OXSR1, ZBTB45, ZBTB44, ZNF585A, ZBTB43, TAX1BP1, ZNF526, DMRTC2, ATP2A3, HGS, RHEB, TCF19, TNK2, ZFHX2, ZFHX3, PLA2G4B, RERE, PLA2G4D, CDC42BPB, ZNF536, SPG7, PXDN, FAM96A, RNPEPL1, RNF187, CPEB1, MMP28, ZBTB37, PARN, SMAP2, MGRN1, SMPDL3B, NUBP2, P4HA3, ZFAT, AGAP1, COL11A2, NR2F2, DPEP2, NT5C, IHH, ZFP36, KCNMA1, ZBTB22, BRF1, PEPD, PDE12, AIFM3, ZC3H7A, MICAL3, ADNP, POLR1B, MMP14, ZNF689, ZSWIM3, ZCCHC14, ZSWIM4, PAPD4, ASH1L, SIAH3, USP22, ZNF276, NKD1, LIMS1, ZFP64, THAP4, COL2A1, ZBTB17, NECAB3, ZNF652, ZFP36L1, MTMR3, ZNF853, SQSTM1, DGKD, PLA2G12A, USP39, DTNB, MT1E, GATAD2B, THAP1, BCL6, UNK, BAZ2B, RASA3, BCKDHA, LMX1B, RNF19A, CSNK2B, HSPG2, ATP1A3, TRIM26, PPP1R10, DGKI, PHF12, CAPN2, BIRC2, ZNF358, CAPN3, TP73, CAPN1, HARBI1, RASSF5, RPS6KA4, CDH16, RPS6KA2, PHF13, ZNF362, SH3RF3, CHN1, PHF21A, DPYD, ZBTB3, CIT, GNA13, GNA14, ZNF581, ZNF584, CNOT8, ZNF580, ZNF827, NR2E3, MCM10, GTF2E1, CIB4, SLC25A25, RABGEF1, CPA2, CTDSP1, ITPK1, OMA1, RNF32, STK25, MTA1, NEBL, KDM2A, CHPF, ATP9B, RNF138, ANKFY1, TRIM39, ARFGAP3, AGFG1, STK11, WT1, ZNF846, CXXC1, PEF1, PDE6B, ECE1, GMIP, ZNF428, ZNF429, RNFT2, RNF167, FGD6, DNAJA4, DNAJA2, OBSCN, ADSSL1, VAV3, EHMT1, SMG6, AKAP8L, FDPS, NR4A2, ITGA1, UBOX5, EHMT2, LHPP, TET2, VAV2, FOXP1, CACNA2D4, SLC25A12, B3GAT1, DNA2, HDAC4, PDZD8, S100B, DOHH, RNF157, HIVEP3, DNMT1, HDHD2, CACNA1H, CACNA1E, SPTA1, ASNA1, CACNA1C, HDAC9, CACNA1B, RAI1, IMPA2, RSF1, MLPH, LYAR, CAD, MAN2B2, PCGF3, TRIM8, GSN, TPP1, ZNF407, ZCCHC9, TPO, EXOG, PDE8B, LOXL2, KDM5A, CDK5RAP1, NDUFS2, LOXL1, ATP8B3, IMPDH2, ZC3H10, NRXN3, LDB3, PRKCH, IRF2BP2, IRF2BP1, CDK2, ARL3, PPM1F, HAGH, MAN2A2, RFWD3, ISCA2, NPTXR, PPM1K, ZMIZ1, CA7, MDM2, PDE9A, CHFR, WIZ, ABLIM2, PRKCZ, GALNT2, ADAMTS14, ADAMTS13, TIPARP, MGMT, GLIS1, CDH1, NR3C1, BRSK1, COMT, PRDM16, ESYT2, CDKAL1, HIC1, PRDM13, JMJD7-PLA2G4B, NPTX1, ZNF709, NUDT8, CYP26B1, GFI1, ING1, ENOSF1, NOX5, ADARB2, DTX3, SAMHD1, PARK2, AMPD2, MAN1C1, COL5A2, COL5A1, RUFY2, CADPS, ADI1, AMDHD2, MPI, SULF2, NME1-NME2, ZBED4, KDM4B, ATP8A2, SP6, LTA4H, JAK2, KDM4A, ALKBH2, ARAP1, ALKBH5, ALKBH4 | 1.401055 | 5.07E-10 | 1.69E-10 |
| UP_KEYWORDS | Zinc | 234 | 15.010 | 7.29E-08 | PNKD, THOP1, SHH, ZFP90, CUL9, ZNF397, ZNF106, RARB, RPP21, CIZ1, POLE, ZNF790, HNF4A, PIAS4, PRDM4, UBR5, TRMT13, ACAP2, ZGLP1, ZNHIT1, ZZZ3, ZNF382, SIVA1, TRIM64C, ZNF132, ENPP2, MYO9A, PXN, ADAP1, NR1H2, ACE, ARIH2, ZNF225, CASZ1, CDADC1, LHX5, RCHY1, DNMT3B, NR1H3, DHX57, KLF5, ZNF529, BMP1, ZC3H18, AGBL4, ZNF621, CREBBP, ANKMY1, AARSD1, SMYD2, ZBTB45, ZBTB44, ZNF585A, TAX1BP1, ZBTB43, ZNF526, DMRTC2, HGS, TCF19, ZFHX2, ZFHX3, RERE, CDC42BPB, ZNF536, SPG7, FAM96A, RNPEPL1, RNF187, CPEB1, MMP28, ZBTB37, SMAP2, MGRN1, SMPDL3B, ZFAT, AGAP1, NR2F2, DPEP2, IHH, ZFP36, ZBTB22, BRF1, ZC3H7A, MICAL3, ADNP, POLR1B, MMP14, ZNF689, ZSWIM3, ZSWIM4, ZCCHC14, ASH1L, SIAH3, USP22, ZNF276, LIMS1, ZFP64, THAP4, SLC39A11, ZBTB17, ZNF652, ZFP36L1, MTMR3, ZNF853, SQSTM1, DGKD, USP39, MT1E, DTNB, GATAD2B, THAP1, BCL6, UNK, BAZ2B, RASA3, LMX1B, RNF19A, CSNK2B, TRIM26, PPP1R10, DGKI, PHF12, ZNF358, BIRC2, TP73, RASSF5, ZNF362, PHF13, CHN1, SH3RF3, PHF21A, ZBTB3, CIT, ZNF581, ZNF584, ZNF580, ZNF827, NR2E3, MCM10, GTF2E1, RABGEF1, CPA2, RNF32, OMA1, MTA1, NEBL, KDM2A, RNF138, ANKFY1, TRIM39, ARFGAP3, AGFG1, WT1, CXXC1, ZNF846, ECE1, ZNF428, GMIP, ZNF429, RNFT2, RNF167, FGD6, DNAJA4, DNAJA2, EHMT1, VAV3, NR4A2, AKAP8L, UBOX5, TET2, EHMT2, VAV2, FOXP1, HDAC4, PDZD8, S100B, RNF157, HIVEP3, CACNA1H, DNMT1, HDAC9, ASNA1, RAI1, RSF1, MLPH, LYAR, CAD, PCGF3, MAN2B2, TRIM8, ZCCHC9, ZNF407, KDM5A, ZC3H10, LDB3, PRKCH, IRF2BP2, IRF2BP1, MAN2A2, HAGH, RFWD3, CA7, ZMIZ1, MDM2, PDE9A, CHFR, WIZ, ABLIM2, PRKCZ, ADAMTS14, ADAMTS13, TIPARP, MGMT, GLIS1, NR3C1, PRDM16, HIC1, PRDM13, ZNF709, GFI1, ING1, ADARB2, DTX3, SAMHD1, PARK2, AMPD2, RUFY2, MPI, ZBED4, KDM4B, SP6, LTA4H, KDM4A, ARAP1 | 1.392456 | 3.51E-05 | 3.19E-06 |
| UP_KEYWORDS | Zinc-finger | 176 | 11.289 | 7.05E-06 | ZNF581, ZNF584, ZNF580, ZNF827, NR2E3, MCM10, GTF2E1, ZFP90, CUL9, RABGEF1, ZNF397, ZNF106, RARB, RNF32, CIZ1, POLE, ZNF790, MTA1, KDM2A, HNF4A, PIAS4, PRDM4, UBR5, TRMT13, ACAP2, ZGLP1, RNF138, ZNHIT1, ZZZ3, ANKFY1, TRIM39, ZNF382, TRIM64C, ARFGAP3, ZNF132, AGFG1, MYO9A, WT1, ADAP1, ZNF846, CXXC1, NR1H2, ARIH2, GMIP, ZNF428, ZNF225, ZNF429, RNFT2, CASZ1, RCHY1, RNF167, FGD6, DNMT3B, DNAJA4, DNAJA2, DHX57, NR1H3, KLF5, ZNF529, VAV3, ZC3H18, CREBBP, ZNF621, AKAP8L, ANKMY1, NR4A2, SMYD2, UBOX5, ZBTB45, VAV2, ZNF585A, ZBTB44, TAX1BP1, ZBTB43, FOXP1, ZNF526, PDZD8, RNF157, HIVEP3, HGS, DNMT1, TCF19, ZFHX2, ZFHX3, RERE, CDC42BPB, ZNF536, RAI1, RSF1, MLPH, LYAR, RNF187, ZBTB37, SMAP2, PCGF3, MGRN1, TRIM8, ZCCHC9, ZNF407, ZFAT, AGAP1, NR2F2, KDM5A, ZFP36, ZBTB22, BRF1, ZC3H10, ZC3H7A, ADNP, PRKCH, IRF2BP2, POLR1B, IRF2BP1, ZNF689, ZSWIM3, ZSWIM4, ZCCHC14, RFWD3, ZMIZ1, ASH1L, SIAH3, MDM2, USP22, CHFR, WIZ, ZNF276, PRKCZ, ZFP64, THAP4, TIPARP, GLIS1, NR3C1, ZBTB17, PRDM16, ZNF652, HIC1, PRDM13, ZFP36L1, MTMR3, ZNF853, ZNF709, SQSTM1, DGKD, USP39, DTNB, GATAD2B, THAP1, BCL6, GFI1, UNK, RASA3, BAZ2B, ING1, RNF19A, DTX3, PPP1R10, TRIM26, PHF12, PARK2, DGKI, BIRC2, ZNF358, RUFY2, RASSF5, ZNF362, PHF13, ZBED4, KDM4B, CHN1, SH3RF3, SP6, PHF21A, ZBTB3, KDM4A, CIT, ARAP1 | 1.380742 | 0.003391 | 2.26E-04 |
| GOTERM_MF_DIRECT | GO:0046872~metal ion binding | 207 | 13.278 | 4.59E-04 | ADCY3, GBGT1, PNKD, FAM20B, THOP1, CIAPIN1, ZFP90, ZNF397, ZNF106, RPP21, ZNF790, OLA1, PITPNM2, PITPNM3, PRDM4, TRMT13, ACAP2, ZNHIT1, FDX1L, PELO, ZNF382, ITGA2B, SIVA1, MGAT5B, GNAI2, ZNF132, ENPP2, NEK2, MYO9A, PPAT, ADAP1, ACE, ZNF225, CASZ1, DNMT3B, NTHL1, DHX57, KLF5, ZNF529, ZC3H18, ZNF621, ANKMY1, AARSD1, SMYD2, ZBTB45, ZBTB44, ZNF585A, TAX1BP1, ZBTB43, ZNF526, DMRTC2, ATP2A3, RHEB, HGS, TNK2, ZFHX2, CDC42BPB, PLA2G4D, ZNF536, PXDN, FAM96A, CPEB1, ZBTB37, SMAP2, PARN, SMPDL3B, NUBP2, ZFAT, AGAP1, COL11A2, DPEP2, NT5C, ZFP36, ZBTB22, KCNMA1, PDE12, AIFM3, ZC3H7A, ADNP, POLR1B, ZNF689, PAPD4, SIAH3, ZNF276, ZFP64, THAP4, COL2A1, ZBTB17, ZNF652, ZFP36L1, MTMR3, ZNF853, DGKD, MT1E, BCL6, UNK, RASA3, BCKDHA, CSNK2B, ATP1A3, TRIM26, PPP1R10, DGKI, ZNF358, TP73, RASSF5, HARBI1, ZNF362, CHN1, ZBTB3, DPYD, CIT, GNA13, ZNF581, GNA14, ZNF584, CNOT8, ZNF580, ZNF827, MCM10, GTF2E1, CTDSP1, OMA1, STK25, CHPF, ANKFY1, ARFGAP3, AGFG1, WT1, ZNF846, PDE6B, ECE1, ZNF428, GMIP, ZNF429, DNAJA4, FGD6, DNAJA2, OBSCN, VAV3, SMG6, ITGA1, FDPS, AKAP8L, VAV2, FOXP1, CACNA2D4, DNA2, B3GAT1, PDZD8, DOHH, HIVEP3, CACNA1H, HDHD2, HDAC9, CACNA1C, ASNA1, MLPH, LYAR, CAD, PCGF3, TPP1, ZNF407, EXOG, PDE8B, CDK5RAP1, NDUFS2, IMPDH2, ZC3H10, NRXN3, PRKCH, IRF2BP2, IRF2BP1, CDK2, ARL3, HAGH, PPM1F, NPTXR, PPM1K, PDE9A, WIZ, PRKCZ, TIPARP, GLIS1, CDKAL1, PRDM16, HIC1, PRDM13, NPTX1, ZNF709, NUDT8, GFI1, ADARB2, PARK2, AMPD2, COL5A2, COL5A1, RUFY2, CADPS, AMDHD2, NME1-NME2, ZBED4, SP6, JAK2, ARAP1, ALKBH5, ALKBH4 | 1.245513 | 0.441257 | 0.109893 |
| **Annotation Cluster 2** | **Enrichment Score: 4.311** |  |  |  |  |  |  |  |
| INTERPRO | IPR011993:Pleckstrin homology-like domain | 62 | 3.977 | 1.31E-06 | PLEKHM2, FERMT3, MCF2L, WBP2, FRMD1, PLEKHB2, VEPH1, RANBP3, AGAP1, SHC3, SHC2, SHC4, AKT2, OSBP2, ANKS1A, DAB2IP, BCR, ARHGEF1, ARHGEF10, TYK2, SBF1, DOK7, ACAP2, CMIP, OSBPL11, SNTG2, TRIOBP, PHLDB1, RABGAP1L, ADAP1, MTMR3, DGKD, NUMB, TBC1D1, RASA3, AFAP1, FGD6, RASA1, OSBPL5, OBSCN, ECT2L, ABR, VAV3, SPTBN5, PTPN3, OSBPL9, HOMER1, VAV2, CADPS, EPS8, RGS3, JAK2, JAK3, SYNGAP1, CIT, PHLDB2, APBB1, ARAP1, PLEKHA2, DNM1, CDC42BPB, SH3BP2 | 1.907112 | 0.002709 | 0.002709 |
| UP_SEQ_FEATURE | domain:PH | 38 | 2.437 | 1.77E-05 | TRIOBP, PHLDB1, PLEKHM2, FERMT3, MCF2L, PLEKHB2, VEPH1, DGKD, AGAP1, RASA3, RASA1, AKT2, OSBPL5, OBSCN, DAB2IP, OSBP2, VAV3, ARHGEF1, BCR, ABR, SPTBN5, OSBPL9, VAV2, CADPS, ADRM1, SBF1, DOK7, ACAP2, CMIP, OSBPL11, CIT, SYNGAP1, PHLDB2, DNM1, PLCXD2, CDC42BPB, SH3BP2, SNTG2 | 2.132839 | 0.06528 | 0.013411 |
| INTERPRO | IPR001849:Pleckstrin homology domain | 39 | 2.502 | 1.84E-04 | PHLDB1, TRIOBP, PLEKHM2, FERMT3, MCF2L, ADAP1, PLEKHB2, VEPH1, DGKD, AGAP1, RASA3, AFAP1, FGD6, RASA1, AKT2, OSBPL5, OBSCN, DAB2IP, OSBP2, VAV3, ARHGEF1, BCR, ABR, SPTBN5, OSBPL9, VAV2, CADPS, SBF1, DOK7, ACAP2, OSBPL11, CIT, SYNGAP1, PHLDB2, DNM1, ARAP1, PLEKHA2, CDC42BPB, SH3BP2 | 1.8902 | 0.317571 | 0.091104 |
| SMART | SM00233:PH | 39 | 2.502 | 0.001332 | PHLDB1, TRIOBP, PLEKHM2, FERMT3, MCF2L, ADAP1, PLEKHB2, VEPH1, DGKD, AGAP1, RASA3, AFAP1, FGD6, RASA1, AKT2, OSBPL5, OBSCN, DAB2IP, OSBP2, VAV3, ARHGEF1, BCR, ABR, SPTBN5, OSBPL9, VAV2, CADPS, SBF1, DOK7, ACAP2, OSBPL11, CIT, SYNGAP1, PHLDB2, DNM1, ARAP1, PLEKHA2, CDC42BPB, SH3BP2 | 1.695997 | 0.440785 | 0.176128 |
| **Annotation Cluster 3** | **Enrichment Score: 4.033** |  |  |  |  |  |  |  |
| UP_SEQ_FEATURE | domain:C2 | 20 | 1.283 | 4.90E-06 | DAB2IP, CAPN5, ABR, BCR, PIK3C2B, UVRAG, WWC1, PRKCH, RIMS3, CADPS, JMJD7-PLA2G4B, RGS3, JMJD7, C2CD4C, CC2D1B, ITCH, SYNGAP1, PLA2G4B, RASA1, PLA2G4D | 3.353951 | 0.018497 | 0.006204 |
| INTERPRO | IPR000008:C2 calcium-dependent membrane targeting | 28 | 1.796 | 1.24E-04 | UVRAG, WWC1, PTH2, ESYT2, PTEN, RIMS3, JMJD7-PLA2G4B, CC2D1B, ITCH, RASA3, RASA1, DAB2IP, CAPN5, BCR, C2CD3, ABR, PIK3C2B, PRKCH, GAK, DOC2A, RGS3, FER1L5, SNRNP200, C2CD4C, SYNGAP1, PLA2G4B, SYTL1, PLA2G4D | 2.228879 | 0.226632 | 0.0821 |
| SMART | SM00239:C2 | 23 | 1.475 | 0.001312 | DAB2IP, CAPN5, ABR, C2CD3, BCR, PIK3C2B, UVRAG, PRKCH, ESYT2, RIMS3, JMJD7-PLA2G4B, DOC2A, RGS3, FER1L5, C2CD4C, CC2D1B, ITCH, SYNGAP1, RASA3, PLA2G4B, SYTL1, RASA1, PLA2G4D | 2.079163 | 0.435883 | 0.248923 |
|  |  |  |  |  |  |  |  |  |
| **Annotation Cluster 4** | **Enrichment Score: 3.515** |  |  |  |  |  |  |  |
| UP_KEYWORDS | ATP-binding | 162 | 10.391 | 4.54E-10 | ADCY3, KIFC2, FAM20B, PRKG1, ACSS2, KIFC3, DDX23, DHX38, CUL9, MLKL, DDX20, ITPK1, EGFR, CIITA, BCR, STK25, CSNK1G2, MYLK3, ABCC11, OLA1, MYLK2, WNK2, ABCC12, MARK3, MYH9, EARS2, MYO18B, MAP4K5, GLUL, CAMK4, ATP9B, NME1, HARS, FLAD1, KIF26A, KIF26B, DNAH10, FGFR3, MVD, STK11, NEK2, MYO9A, UBE2D2, SBK1, SBK3, LMTK2, NAT10, STK19, DHX57, DDX42, DHX9, OBSCN, ABCB9, MYO1C, MYO1E, SPHK1, MYO1D, TAOK3, ATP11A, OXSR1, KIF3C, DDX4, ABCG8, DNA2, ABCG5, PLK2, PSMC4, ATP2A3, ULK1, MYH11, GRK6, GRK7, SLFNL1, TNK2, HSPA13, GRK5, FUK, PIP4K2A, ASNA1, CLCN7, CACNA1B, CDC42BPB, CDK19, ABCF1, KIF22, SPG7, PRKAG2, CAD, ACSF3, CAMKK1, NOD2, ATAD3A, NUBP2, NARS2, SLC22A4, EEF2K, PAK1, DYNC1H1, ATP8B3, KIAA0232, AKT2, SRPK2, PIK3C2B, NADSYN1, PRKCH, CDK9, DARS2, CDK4, DAPK2, DAPK3, UBE2B, CDK2, GAK, MCM6, MAST3, PANK4, TYK2, UBE2O, TRAP1, STYK1, PAPD4, RRM1, SNRNP200, UBE2W, SMARCAD1, PRKCZ, SKIV2L, CAMK2G, DNAH2, BRSK1, VARS, IARS, GALK2, STK40, DGKD, DHX15, TOR1B, CERK, ACSL3, HSPA8, MARS, AATK, MAK, MSH4, ATP1A3, DGKI, TEX14, RPS6KA4, NME1-NME2, HELB, RPS6KA2, ATP8A2, ABCC3, TEP1, CHTF18, UCKL1, JAK2, JAK3, AACS, IPPK, CIT, PAICS | 1.62724 | 2.19E-07 | 3.64E-08 |
| UP_KEYWORDS | Nucleotide-binding | 195 | 12.508 | 1.47E-09 | ADCY3, KIFC2, FAM20B, PRKG1, KIFC3, DHX38, MTG1, CUL9, RAPGEF4, BCR, RAN, MYLK3, ABCC11, MYLK2, OLA1, WNK2, ABCC12, MARK3, MYH9, MYO18B, GLUL, CAMK4, NME1, HARS, KIF26A, KIF26B, DNAH10, GNAI2, NEK2, RAB40C, MYO9A, RAB40B, RAC3, NAT10, DHX57, DHX9, SPHK1, TAOK3, ATP11A, OXSR1, VDAC2, KIF3C, DDX4, ABCG8, ABCG5, ATP2A3, GRK6, RHEB, SLFNL1, GRK7, TNK2, FUK, GRK5, HSPA13, CLCN7, CDC42BPB, ABCF1, SPG7, CAMKK1, NOD2, ATAD3A, NUBP2, SLC22A4, EEF2K, DYNC1H1, AGAP1, NT5C, SRPK2, PIK3C2B, RAB4B, DARS2, MAST3, TRAP1, STYK1, PAPD4, RRM1, ARL4C, SKIV2L, GALK2, STK40, DGKD, DHX15, RAB11B, RAB11A, CERK, ACSL3, MARS, AATK, ATP1A3, DGKI, TEX14, RPS6KA4, HELB, RPS6KA2, RAB35, ABCC3, CHTF18, DPYD, CIT, AACS, PAICS, DNM1, GNA13, GNA14, CNGB1, ACSS2, DDX23, MLKL, DDX20, ITPK1, CIITA, EGFR, STK25, CSNK1G2, EARS2, MAP4K5, ATP9B, FLAD1, FGFR3, MVD, STK11, UBE2D2, SBK1, SBK3, GFM1, LMTK2, TUBE1, STK19, DDX42, OBSCN, ADSSL1, ABCB9, MYO1C, MYO1E, MYO1D, DNA2, PSMC4, PLK2, ULK1, PRKAR1B, MYH11, ASNA1, PIP4K2A, CACNA1B, CDK19, KIF22, ATL1, PRKAG2, ATL3, CAD, ACSF3, NARS2, PAK1, RHOF, KIAA0232, ATP8B3, AKT2, RABL3, NADSYN1, PRKCH, CDK9, DAPK2, CDK4, DAPK3, UBE2B, CDK2, GAK, MCM6, ARL3, PANK4, TYK2, UBE2O, SNRNP200, HHAT, UBE2W, NKIRAS1, SMARCAD1, PRKCZ, CAMK2G, DNAH2, BRSK1, VARS, IARS, TOR1B, HSPA8, MAK, MSH4, RABL2A, NME1-NME2, ATP8A2, TEP1, UCKL1, JAK2, JAK3, IPPK | 1.52381 | 7.11E-07 | 1.02E-07 |
| GOTERM_MF_DIRECT | GO:0005524~ATP binding | 172 | 11.032 | 7.92E-07 | ADCY3, KIFC2, ATP1B1, FAM20B, PRKG1, ACSS2, KIFC3, DDX23, DHX38, CUL9, MLKL, DDX20, ITPK1, EGFR, CIITA, TWF2, BCR, STK25, CSNK1G2, MYLK3, ABCC11, OLA1, MYLK2, WNK2, ABCC12, MARK3, MYH9, EARS2, MYO18B, MAP4K5, GLUL, CAMK4, ATP9B, NME1, HARS, FLAD1, KIF26A, KIF26B, DNAH10, FGFR3, MVD, STK11, NEK2, MYO9A, UBE2D2, SBK1, SBK3, MYO15B, LMTK2, NAT10, STK19, DNAJA4, DNAJA2, DHX57, DDX42, DHX9, OBSCN, ABCB9, SMCHD1, MYO1C, MYO1E, SPHK1, MYO1D, TAOK3, ATP11A, DALRD3, OXSR1, AARSD1, KIF3C, DDX4, ABCG8, DNA2, ABCG5, PLK2, PSMC4, ATP2A3, ULK1, GRK6, MYH11, GRK7, SLFNL1, TNK2, HSPA13, FUK, GRK5, PIP4K2A, ASNA1, CLCN7, CACNA1B, CDC42BPB, CDK19, ABCF1, KIF22, SPG7, PRKAG2, CAD, ACSF3, CAMKK1, NOD2, ATAD3A, NUBP2, NARS2, SLC22A4, EEF2K, PAK1, DYNC1H1, ATP8B3, KIAA0232, AKT2, SRPK2, PIK3C2B, NADSYN1, PRKCH, CDK9, DARS2, CDK4, DAPK2, DAPK3, UBE2B, CDK2, GAK, MCM6, MAST3, PANK4, TYK2, UBE2O, TRAP1, STYK1, PAPD4, RRM1, SNRNP200, UBE2W, SMARCAD1, PRKCZ, SKIV2L, CAMK2G, DNAH2, BRSK1, VARS, ITM2C, IARS, GALK2, STK40, DGKD, DHX15, TOR1B, CERK, ACSL3, HSPA8, MARS, AATK, ALPK1, MAK, MSH4, ATP1A3, DGKI, TEX14, RPS6KA4, NME1-NME2, HELB, RPS6KA2, ATP8A2, ABCC3, TEP1, CHTF18, UCKL1, JAK2, JAK3, AACS, IPPK, CIT, PAICS | 1.432273 | 0.001005 | 5.03E-04 |
| UP_SEQ_FEATURE | nucleotide phosphate-binding region:ATP | 109 | 6.992 | 1.48E-05 | KIFC2, PRKG1, KIFC3, DHX38, DDX23, CUL9, DDX20, ITPK1, CIITA, EGFR, STK25, CSNK1G2, MYLK3, OLA1, MYLK2, WNK2, MARK3, MYH9, EARS2, MYO18B, MAP4K5, CAMK4, KIF26A, KIF26B, DNAH10, FGFR3, STK11, NEK2, MYO9A, SBK1, LMTK2, NAT10, DDX42, DHX57, OBSCN, DHX9, ABCB9, MYO1C, MYO1E, MYO1D, TAOK3, OXSR1, DDX4, KIF3C, DNA2, ABCG5, PLK2, PSMC4, ULK1, MYH11, GRK6, GRK7, SLFNL1, TNK2, GRK5, FUK, ASNA1, CLCN7, CACNA1B, CDC42BPB, CDK19, KIF22, SPG7, ACSF3, CAMKK1, NOD2, ATAD3A, NUBP2, SLC22A4, EEF2K, PAK1, DYNC1H1, KIAA0232, AKT2, SRPK2, NADSYN1, CDK9, PRKCH, DAPK2, CDK4, DAPK3, CDK2, MCM6, MAST3, TYK2, STYK1, SNRNP200, SMARCAD1, PRKCZ, CAMK2G, SKIV2L, BRSK1, DNAH2, GALK2, STK40, DHX15, TOR1B, AATK, MAK, MSH4, TEX14, RPS6KA4, RPS6KA2, TEP1, UCKL1, CHTF18, JAK2, JAK3, CIT | 1.507928 | 0.054817 | 0.013995 |
| UP_KEYWORDS | Serine/threonine-protein kinase | 51 | 3.271 | 5.29E-05 | CDK19, PRKG1, CAMKK1, EEF2K, PAK1, ADCK5, AKT2, SRPK2, BCR, STK25, CSNK1G2, MYLK3, CDK9, MYLK2, PRKCH, WNK2, MARK3, DAPK2, CDK4, DAPK3, CDK2, GAK, MAST3, MAP4K5, CAMK4, PRKCZ, STK11, NEK2, CAMK2G, BRSK1, STK40, SBK1, SBK3, LMTK2, STK19, AATK, OBSCN, ALPK1, MAK, TAOK3, OXSR1, RPS6KA4, PLK2, ULK1, RPS6KA2, GRK6, GRK7, TNK2, GRK5, CIT, CDC42BPB | 1.813182 | 0.025176 | 0.001499 |
| UP_KEYWORDS | Kinase | 82 | 5.260 | 6.48E-05 | CDK19, FAM20B, PRKAG2, PRKG1, CAMKK1, PACSIN1, EEF2K, PAK1, ITPK1, ADCK5, AKT2, EGFR, CIITA, SRPK2, BCR, STK25, CSNK1G2, PIK3C2B, MYLK3, CDK9, MYLK2, PRKCH, WNK2, DAPK2, CDK4, MARK3, DAPK3, CDK2, GAK, TYK2, PANK4, MAST3, STYK1, MAP4K5, CAMK4, NME1, CDK2AP2, NRGN, PRKCZ, FGFR3, STK11, NEK2, CAMK2G, BRSK1, GALK2, SBK1, STK40, DGKD, SBK3, LMTK2, CERK, STK19, AATK, OBSCN, ALPK1, MAK, TAOK3, SPHK1, CSNK2B, AKAP8L, OXSR1, DGKI, RPS6KA4, PLK2, EPS8, NME1-NME2, ULK1, RPS6KA2, PRKAR1B, GRK6, UCKL1, HGS, GRK7, JAK2, TNK2, JAK3, FUK, GRK5, PIP4K2A, CIT, IPPK, CDC42BPB | 1.558799 | 0.030736 | 0.001733 |
| UP_SEQ_FEATURE | binding site:ATP | 63 | 4.041 | 5.56E-04 | CDK19, SPG7, PRKG1, ACSF3, CAMKK1, PAK1, ITPK1, AKT2, EGFR, SRPK2, STK25, CSNK1G2, MYLK3, MYLK2, PRKCH, CDK9, WNK2, MARK3, CDK4, DAPK2, DAPK3, CDK2, EARS2, TYK2, MAST3, TRAP1, STYK1, MAP4K5, CAMK4, NME1, PRKCZ, FGFR3, STK11, NEK2, CAMK2G, BRSK1, VARS, IARS, STK40, SBK1, LMTK2, AATK, MARS, OBSCN, MAK, TAOK3, OXSR1, TEX14, RPS6KA4, PLK2, NME1-NME2, ATP2A3, ULK1, RPS6KA2, GRK6, GRK7, JAK2, TNK2, JAK3, GRK5, CIT, ASNA1, CDC42BPB | 1.552555 | 0.879621 | 0.190801 |
| INTERPRO | IPR011009:Protein kinase-like domain | 61 | 3.913 | 0.001305 | CDK19, PRKG1, CAMKK1, EEF2K, MLKL, PAK1, ADCK5, AKT2, EGFR, SRPK2, STK25, CSNK1G2, MYLK3, PIK3C2B, MYLK2, PRKCH, CDK9, WNK2, MARK3, CDK4, DAPK2, DAPK3, CDK2, GAK, TYK2, MAST3, STYK1, MAP4K5, CAMK4, PRKCZ, FGFR3, STK11, NEK2, CAMK2G, TRRAP, BRSK1, STK40, SBK1, SBK3, LMTK2, KNDC1, AATK, OBSCN, ALPK1, MAK, TAOK3, OXSR1, TEX14, RPS6KA4, PLK2, ULK1, RPS6KA2, GRK6, GRK7, JAK2, TNK2, JAK3, GRK5, CIT, ACAD10, CDC42BPB | 1.508856 | 0.933095 | 0.362848 |
| GOTERM_MF_DIRECT | GO:0004674~protein serine/threonine kinase activity | 48 | 3.079 | 0.0016 | CDK19, PRKCZ, NEK2, STK11, CAMK2G, BRSK1, PRKG1, CAMKK1, STK40, SBK1, SQSTM1, SBK3, EEF2K, LMTK2, PAK1, STK19, ADCK5, AATK, AKT2, OBSCN, SRPK2, ALPK1, BCR, CSNK1G2, MAK, TAOK3, CSNK2B, PRKCH, CDK9, OXSR1, WNK2, CDK4, DAPK2, MARK3, DAPK3, CDK2, GAK, MAST3, MAP4K5, RPS6KA4, NME1-NME2, PLK2, RPS6KA2, ULK1, TNK2, GRK5, CIT, CDC42BPB | 1.589249 | 0.868935 | 0.224314 |
| INTERPRO | IPR008271:Serine/threonine-protein kinase, active site | 39 | 2.502 | 0.002702 | CDK19, PRKCZ, NEK2, STK11, CAMK2G, BRSK1, PRKG1, CAMKK1, STK40, SBK1, SBK3, PAK1, AKT2, OBSCN, SRPK2, CSNK1G2, MAK, MYLK3, TAOK3, PRKCH, MYLK2, CDK9, WNK2, DAPK2, CDK4, MARK3, DAPK3, CDK2, GAK, MAST3, RPS6KA4, PLK2, CAMK4, RPS6KA2, ULK1, GRK6, GRK7, CIT, CDC42BPB | 1.641808 | 0.996314 | 0.428979 |
| INTERPRO | IPR017441:Protein kinase, ATP binding site | 45 | 2.886 | 0.003666 | CDK19, PRKCZ, FGFR3, STK11, CAMK2G, BRSK1, PRKG1, CAMKK1, SBK3, LMTK2, PAK1, AATK, AKT2, EGFR, OBSCN, SRPK2, STK25, CSNK1G2, MAK, MYLK3, TAOK3, PRKCH, MYLK2, CDK9, OXSR1, CDK4, DAPK2, MARK3, DAPK3, CDK2, TYK2, MAP4K5, RPS6KA4, PLK2, CAMK4, RPS6KA2, ULK1, GRK6, GRK7, JAK2, TNK2, JAK3, GRK5, CIT, CDC42BPB | 1.551315 | 0.999503 | 0.469442 |
| INTERPRO | IPR000719:Protein kinase, catalytic domain | 54 | 3.464 | 0.005273 | CDK19, PRKG1, CAMKK1, MLKL, PAK1, AKT2, EGFR, SRPK2, STK25, CSNK1G2, MYLK3, MYLK2, PRKCH, CDK9, WNK2, MARK3, CDK4, DAPK2, DAPK3, CDK2, GAK, TYK2, MAST3, STYK1, MAP4K5, CAMK4, PRKCZ, FGFR3, STK11, NEK2, CAMK2G, BRSK1, STK40, SBK1, SBK3, LMTK2, AATK, OBSCN, MAK, TAOK3, OXSR1, TEX14, RPS6KA4, PLK2, ULK1, RPS6KA2, GRK6, GRK7, JAK2, TNK2, JAK3, GRK5, CIT, CDC42BPB | 1.456388 | 0.999982 | 0.474877 |
| GOTERM_BP_DIRECT | GO:0046777~protein autophosphorylation | 24 | 1.539 | 0.00872 | EGFR, BCR, FGFR3, STK25, CSNK1G2, STK11, MAK, NEK2, CAMK2G, TAOK3, MYLK2, CAD, WNK2, DAPK2, DAPK3, CAMK4, NME1-NME2, ULK1, EEF2K, LMTK2, GRK7, JAK2, PAK1, GRK5 | 1.768355 | 1 | 0.931903 |
| GOTERM_BP_DIRECT | GO:0006468~protein phosphorylation | 51 | 3.271 | 0.010987 | CDK19, PRKAG2, PRKG1, CAMKK1, MLKL, PAK1, ADCK5, SRPK2, BCR, STK25, CSNK1G2, MYLK3, CDK9, PRKCH, WNK2, CDK4, DAPK2, DAPK3, GAK, TYK2, MAP4K5, CAMK4, WNT11, FASTKD5, PRKCZ, STK11, NEK2, BRSK1, STK40, SBK1, SQSTM1, SBK3, LMTK2, STK19, AATK, OBSCN, ALPK1, MAK, TAOK3, CSNK2B, OXSR1, TEX14, RPS6KA4, PLK2, ULK1, PRKAR1B, GRK6, JAK2, JAK3, CIT, CDC42BPB | 1.417398 | 1 | 0.94699 |
| UP_SEQ_FEATURE | domain:Protein kinase | 48 | 3.079 | 0.021462 | CDK19, PRKCZ, FGFR3, NEK2, STK11, CAMK2G, BRSK1, PRKG1, CAMKK1, STK40, SBK1, LMTK2, MLKL, PAK1, ADCK5, AKT2, AATK, EGFR, SRPK2, STK25, CSNK1G2, MAK, MYLK3, TAOK3, PRKCH, MYLK2, CDK9, OXSR1, WNK2, CDK4, DAPK2, MARK3, DAPK3, CDK2, GAK, MAST3, STYK1, MAP4K5, TEX14, PLK2, CAMK4, ULK1, GRK6, GRK7, TNK2, GRK5, CIT, CDC42BPB | 1.380874 | 1 | 0.853514 |
| SMART | SM00220:S_TKc | 43 | 2.758 | 0.028398 | CDK19, PRKCZ, NEK2, STK11, CAMK2G, BRSK1, PRKG1, CAMKK1, STK40, SBK1, SBK3, PAK1, AKT2, OBSCN, SRPK2, STK25, CSNK1G2, MAK, MYLK3, TAOK3, PRKCH, MYLK2, CDK9, OXSR1, WNK2, DAPK2, CDK4, MARK3, DAPK3, CDK2, GAK, MAST3, MAP4K5, RPS6KA4, PLK2, CAMK4, RPS6KA2, ULK1, GRK6, GRK7, GRK5, CIT, CDC42BPB | 1.375113 | 0.999996 | 0.648915 |
| UP_SEQ_FEATURE | active site:Proton acceptor | 62 | 3.977 | 0.042894 | CDK19, PXDN, CPT2, PRKG1, CAMKK1, TPO, EXOG, PAK1, AKT2, EGFR, SRPK2, STK25, CSNK1G2, MYLK3, MYLK2, PRKCH, CDK9, WNK2, MARK3, CDK4, DAPK2, DAPK3, CDK2, GAK, TYK2, MAST3, STYK1, MAP4K5, CAMK4, H6PD, RRM1, TXNRD2, PRKCZ, FGFR3, STK11, NEK2, CAMK2G, BRSK1, STK40, SBK1, LMTK2, ALDH4A1, AATK, OBSCN, MAK, TAOK3, OXSR1, SULT6B1, B3GAT1, RPS6KA4, PLK2, ULK1, RPS6KA2, GRK6, GRK7, JAK2, TNK2, JAK3, GRK5, CIT, RDH14, CDC42BPB | 1.268712 | 1 | 0.94092 |
| GOTERM_MF_DIRECT | GO:0004672~protein kinase activity | 36 | 2.309 | 0.131648 | CDK19, PRKCZ, NEK2, STK11, CAD, BRSK1, EEF2K, MLKL, PAK1, AATK, AKT2, EGFR, OBSCN, SRPK2, STK25, CSNK1G2, MAK, TAOK3, PRKCH, CDK9, DAPK2, CDK4, DAPK3, CDK2, GAK, TYK2, STYK1, MAP4K5, TEX14, CAMK4, RPS6KA2, JAK2, TNK2, CIT, FASTKD5, CDC42BPB | 1.248379 | 1 | 0.95683 |
| **Annotation Cluster 5** | **Enrichment Score: 2.619** |  |  |  |  |  |  |  |
| UP_KEYWORDS | Iron-sulfur | 13 | 0.834 | 9.63E-04 | DNA2, AIFM3, ISCA2, NUBP2, POLE, FDX1L, DPYD, CDK5RAP1, CDKAL1, NTHL1, NDUFS2, CIAPIN1, PPAT | 3.027303 | 0.371338 | 0.017694 |
| GOTERM_MF_DIRECT | GO:0051539~4 iron, 4 sulfur cluster binding | 11 | 0.706 | 0.001464 | NFU1, DNA2, ISCA2, NUBP2, POLE, DPYD, CDK5RAP1, CDKAL1, NTHL1, NDUFS2, PPAT | 3.260483 | 0.844272 | 0.266509 |
| UP_KEYWORDS | 4Fe-4S | 9 | 0.577 | 0.004001 | DNA2, NUBP2, POLE, DPYD, CDK5RAP1, CDKAL1, NTHL1, NDUFS2, PPAT | 3.398635 | 0.855175 | 0.058595 |
| UP_SEQ_FEATURE | metal ion-binding site:Iron-sulfur (4Fe-4S) | 6 | 0.385 | 0.00595 | NUBP2, CDK5RAP1, CDKAL1, NTHL1, NDUFS2, PPAT | 4.853365 | 1 | 0.56894 |
| **Annotation Cluster 6** | **Enrichment Score: 2.536** |  |  |  |  |  |  |  |
| UP_KEYWORDS | Nucleus | 471 | 30.211 | 4.98E-09 | SCAF1, ASCC1, INTS1, MXI1, CIAPIN1, DHX38, INTS5, MED26, CDCA2, ZNF397, ZNF106, SMOX, TIGD5, ZNF790, HMG20A, MYO18B, DCAF6, AQR, DCAF7, SPAG7, HNF4A, NME1, ZNF783, ZGLP1, TGIF1, ZNHIT1, ZZZ3, PELO, CMIP, ZNF382, ZNF132, NFKBIA, TEN1, XAB2, ADAP1, NPHP4, ARIH2, ZNF225, DUSP16, CASZ1, NAT10, RCHY1, NTHL1, DNMT3B, DHX9, CCDC86, ZNF529, ZC3H18, SPHK1, ZNF621, TMEM120B, NUF2, SFMBT1, ZNF585A, SFMBT2, ZNF526, DMRTC2, RGS3, TNK2, POP5, FOXI1, RERE, PLCXD2, KCNH1, TAF1C, ZNF536, ELF1, ELF2, BACH2, RNF187, MGRN1, NUBP2, FOXB2, DPP9, HSF4, BATF3, SRPK2, BRF1, ZC3H7A, PIK3C2B, EXOSC5, MICAL3, ADNP, TLE4, EXOSC3, ZNF689, TAF11, FMN1, BHMG1, PAPD4, NAB2, NAIF1, BRDT, USP22, THOC5, EEF1D, ZNF276, SKIV2L, AZIN1, ZNF652, NUMA1, REXO4, NUP210, USP39, DHX15, TBC1D1, FOXD1, SIM1, TXNL4B, LMX1B, PPP1R10, DGKI, PHF12, ZNF358, TP73, DOT1L, HARBI1, RPS6KA4, PHF13, RPS6KA2, ZNF362, CHTF18, ZBTB3, USP48, GNA13, MMS19, ZNF581, ZNF584, CNOT8, MAU2, ZNF580, NR2E3, GTF2E1, MIER2, DDX23, BHLHA15, DNAJC7, CTDSP1, DDX20, DND1, EGFR, RBFOX3, ENC1, HNRNPA2B1, MTA1, ARID1B, SIX6, HSPB9, KDM2A, CAMTA1, ACHE, CAMTA2, STK11, UBE2V1, MEIS1, KNOP1, CXXC1, OAZ2, HNRNPM, NIPBL, ZNF429, CERS2, TEF, STK19, DDX42, MUC1, GINS2, TAF4, EHMT1, SMG6, EPAS1, MYO1C, TAF8, NR4A2, AKAP8L, ARID3B, TEAD1, EHMT2, HDAC4, DNA2, DNASE1, HNRNPH3, R3HDM2, S100B, SMARCC2, HIVEP3, HDAC9, DNAJB6, IER2, KIF22, MAD1L1, RAI1, FOSL2, RSF1, ARID4A, LYAR, WWC1, EDC4, CAD, NDUFAF3, HEY1, RTF1, ZNF407, ZCCHC9, CDK5RAP3, LRWD1, KDM5A, LOXL2, AHNAK, IMPDH2, SSBP3, ARID5B, CDK9, IRF2BP2, IRF2BP1, CDK4, CDK2, ARL3, HOXC10, RFWD3, SNRNP200, LCOR, CHFR, WIZ, IRX3, BCLAF1, FKBP5, NR3C1, BRSK1, PRDM16, IVNS1ABP, APLP2, HIC1, PRDM13, REL, TSC22D4, TOR1B, MYCBP, MLXIP, NFATC2, HSPA8, NFATC1, ADARB2, MAK, SAMHD1, CDC25C, VSX2, ADI1, ZBED4, KDM4B, CABIN1, UCKL1, JAK2, KDM4A, IPPK, ALKBH2, ALKBH5, ALKBH4, CDX1, PNMA2, PNKD, SNIP1, LEMD2, ZFP90, RARB, SUPT5H, RPP25, LYRM1, PRAME, RPP21, CIZ1, RAN, LYRM4, POLE, OLA1, TAF6L, TBR1, PPARGC1B, RFC3, PRDM4, PIAS4, CEP350, CAMK4, UBR5, PARP14, PDE4DIP, SIVA1, LMNB1, NEK2, ELL, DAG1, IGF2BP1, NR1H2, AHRR, ASB10, LHX5, NR1H3, KLF5, UFSP2, CREBBP, CIDEA, SKI, SMYD2, DONSON, ZBTB45, ZBTB44, ZBTB43, PTPN11, NOTCH2, ATP2A3, DMTF1, NOP16, TCF19, GRK5, ZFHX2, SNRNP27, CHAF1A, ZFHX3, NCOR2, ALG14, ABCF1, PTEN, ZBTB37, CAMKK1, DIP2A, PARN, AES, RRP1B, ANKRD11, RBM47, ZFAT, NR2F2, FANCA, AGPAT3, FANCC, ZFP36, ZBTB22, TBX15, POLR1E, VEZT, SPEN, POLR1B, ECSIT, ASH1L, TBX18, GADD45A, TRIOBP, ZFP64, SLC39A11, SFN, ZBTB17, SEC14L2, RPP14, ZFP36L1, STAT4, ATXN2L, STK40, SQSTM1, CD2BP2, GATAD2B, BCL3, THAP1, CC2D1B, AATF, BCL6, HBP1, BAZ2B, BCAS3, BCL9, EIF4ENIF1, IPO11, MACROD2, MACROD1, BIRC2, SUGP2, MEOX2, HELB, FYTTD1, NHLH2, PHF21A, APBB1, LOR, TMEM189-UBE2V1, CCDC85B, ZNF827, EIF5A, CBX3, MCM10, RAD21, ITCH, ATOH8, SS18L1, CIITA, TOR1AIP1, FOXN3, ELL2, ADRM1, CELF3, SNRPB, AGFG1, GRB2, TRRAP, WT1, ZNF846, DAZAP1, FOXQ1, PPL, AXIN1, SREBF1, PTPN6, TMEM97, TBL3, HTT, UBOX5, LHPP, MED13L, FOXP1, SREBF2, HOXB7, PSMC4, DNMT1, PIP4K2A, ASNA1, PLEKHA2, FOXK1, PCGF3, RANBP3, AKT2, SOX13, UBE2B, DAPK3, UBN1, MCM6, UBE2O, SBF1, TPPP, LARP7, ZMIZ1, MDM2, UBE2W, CUEDC2, SMARCAD1, MGMT, GLIS1, CTNND2, HCFC2, UBE3C, NPAS1, ZNF709, POU2F2, GFI1, TNPO1, TINF2, ING1, BAHD1, TNPO3, KAT2A, CENPN, CENPL, PARK2, FAM60A, RGS14, RUFY2, ATXN1, NME1-NME2, TMEM43, ANXA11, SP6, IRF2, TEP1, NFIC | 1.254937 | 2.40E-06 | 3.00E-07 |
| UP_KEYWORDS | Transcription regulation | 208 | 13.342 | 4.89E-04 | CDX1, ASCC1, MXI1, MED26, ZFP90, ZNF397, RARB, SUPT5H, PRAME, ZNF790, HMG20A, TAF6L, TBR1, PPARGC1B, HNF4A, PIAS4, PRDM4, ZNF783, PARP14, ZGLP1, TGIF1, ZZZ3, ZNF382, ZNF132, ELL, NR1H2, AHRR, ZNF225, CASZ1, LHX5, NR1H3, KLF5, ZNF529, ZNF621, CREBBP, CIDEA, SMYD2, ZBTB45, SFMBT1, ZBTB44, ZNF585A, ZBTB43, ZNF526, NOTCH2, DMRTC2, DMTF1, TCF19, ZFHX2, FOXI1, ZFHX3, NCOR2, RERE, ZNF536, TAF1C, ELF1, BACH2, ELF2, ZBTB37, AES, FOXB2, ZFAT, HSF4, NR2F2, ZBTB22, BATF3, TBX15, BRF1, ADNP, TLE4, SPEN, ZNF689, BHMG1, TAF11, NAB2, ASH1L, BRDT, USP22, VOPP1, EEF1D, TBX18, ZNF276, ZFP64, ZBTB17, SEC14L2, ZNF652, STAT4, BCL3, GATAD2B, BCL6, HBP1, CC2D1B, THAP1, BAZ2B, BCAS3, FOXD1, SIM1, LMX1B, PHF12, ZNF358, BIRC2, TP73, MEOX2, ZNF362, PHF21A, NHLH2, ZBTB3, APBB1, MMS19, ZNF581, ZNF584, CNOT8, CCDC85B, ZNF580, ZNF827, CBX3, NR2E3, GTF2E1, MIER2, BHLHA15, ATOH8, SS18L1, CIITA, MTA1, ARID1B, FOXN3, ELL2, KDM2A, CAMTA1, CAMTA2, TRRAP, WT1, CXXC1, ZNF846, FOXQ1, ZNF429, TEF, SREBF1, TAF4, EPAS1, TAF8, NR4A2, AKAP8L, TEAD1, ARID3B, MED13L, FOXP1, SREBF2, HDAC4, HOXB7, SMARCC2, HIVEP3, DNMT1, HDAC9, RSF1, FOSL2, ARID4A, FOXK1, WWC1, BZW1, PCGF3, HEY1, RTF1, ZNF407, KDM5A, LOXL2, SSBP3, SOX13, ARID5B, CDK9, IRF2BP2, IRF2BP1, DAPK3, HOXC10, ZMIZ1, LCOR, IRX3, BCLAF1, SBNO2, GLIS1, CTNND2, NR3C1, PRDM16, HIC1, PRDM13, NPAS1, REL, ZNF709, TSC22D4, POU2F2, MLXIP, MYCBP, GFI1, NFATC2, HSPA8, BAHD1, NFATC1, KAT2A, MAK, PARK2, VSX2, ATXN1, NME1-NME2, KDM4B, IRF2, SP6, KDM4A, NFIC, ALKBH4 | 1.246231 | 0.210066 | 0.010661 |
| UP_KEYWORDS | Transcription | 213 | 13.663 | 5.00E-04 | CDX1, ASCC1, MXI1, MED26, ZFP90, ZNF397, RARB, SUPT5H, PRAME, ZNF790, HMG20A, TAF6L, TBR1, PPARGC1B, HNF4A, PIAS4, PRDM4, ZNF783, PARP14, ZGLP1, TGIF1, ZZZ3, ZNF382, ZNF132, ELL, XAB2, NR1H2, AHRR, ZNF225, CASZ1, LHX5, NR1H3, KLF5, ZNF529, ZNF621, CREBBP, CIDEA, SMYD2, ZBTB45, SFMBT1, ZBTB44, ZNF585A, ZBTB43, ZNF526, NOTCH2, DMRTC2, DMTF1, TCF19, ZFHX2, ZFHX3, FOXI1, RERE, NCOR2, ZNF536, TAF1C, ELF1, BACH2, ELF2, ZBTB37, AES, FOXB2, ZFAT, HSF4, NR2F2, ZBTB22, BATF3, TBX15, BRF1, POLR1E, ADNP, TLE4, SPEN, POLR1B, ZNF689, BHMG1, TAF11, NAB2, ASH1L, BRDT, USP22, VOPP1, EEF1D, TBX18, ZNF276, ZFP64, ZBTB17, SEC14L2, ZNF652, STAT4, BCL3, GATAD2B, BCL6, HBP1, CC2D1B, THAP1, BAZ2B, BCAS3, FOXD1, SIM1, LMX1B, PHF12, ZNF358, BIRC2, TP73, MEOX2, ZNF362, PHF21A, NHLH2, ZBTB3, APBB1, MMS19, ZNF581, ZNF584, CNOT8, CCDC85B, ZNF580, ZNF827, CBX3, NR2E3, GTF2E1, MIER2, BHLHA15, ATOH8, SS18L1, CIITA, MTA1, ARID1B, FOXN3, ELL2, KDM2A, CAMTA1, CAMTA2, TRRAP, MEIS1, WT1, CXXC1, ZNF846, FOXQ1, ZNF429, TEF, SREBF1, TAF4, EPAS1, TAF8, NR4A2, AKAP8L, TEAD1, ARID3B, MED13L, FOXP1, SREBF2, HDAC4, HOXB7, SMARCC2, HIVEP3, DNMT1, HDAC9, RSF1, FOSL2, ARID4A, FOXK1, WWC1, BZW1, PCGF3, HEY1, RTF1, ZCCHC9, ZNF407, KDM5A, LOXL2, SSBP3, SOX13, ARID5B, CDK9, IRF2BP2, IRF2BP1, DAPK3, HOXC10, ZMIZ1, LCOR, IRX3, BCLAF1, SBNO2, GLIS1, CTNND2, NR3C1, PRDM16, HIC1, PRDM13, NPAS1, REL, ZNF709, TSC22D4, POU2F2, MLXIP, MYCBP, GFI1, NFATC2, HSPA8, BAHD1, NFATC1, KAT2A, MAK, PARK2, VSX2, ATXN1, NME1-NME2, KDM4B, IRF2, SP6, KDM4A, NFIC, ALKBH4 | 1.241064 | 0.214343 | 0.010001 |
| UP_KEYWORDS | DNA-binding | 164 | 10.520 | 0.076999 | ZNF581, ZNF584, CDX1, ZNF580, ZNF827, NR2E3, MXI1, MCM10, BHLHA15, ZFP90, ZNF397, TIGD5, RARB, DDX20, ATOH8, POLE, ZNF790, MTA1, HMG20A, ARID1B, TBR1, SIX6, FOXN3, RFC3, KDM2A, HNF4A, PIAS4, PRDM4, ZNF783, ZGLP1, RNF138, ZZZ3, TGIF1, ZNF382, ZNF132, AGFG1, TEN1, MEIS1, WT1, ZNF846, CXXC1, NR1H2, FOXQ1, AHRR, ZNF225, ZNF429, CERS2, TEF, CASZ1, LHX5, STRBP, DNMT3B, NR1H3, SREBF1, KLF5, DHX9, ZNF529, SMG6, EPAS1, ZNF621, NR4A2, TEAD1, ARID3B, TET2, ZBTB45, ZBTB44, ZBTB43, FOXP1, SREBF2, ZNF526, DNA2, DMRTC2, HOXB7, DMTF1, DNMT1, ZFHX2, ZFHX3, FOXI1, NCOR2, IER2, TAF1C, ZNF536, KIF22, ELF1, ELF2, FOSL2, BACH2, FOXK1, ZBTB37, HEY1, RTF1, ZNF407, FOXB2, ZFAT, HSF4, NR2F2, IMPDH2, BATF3, ZFP36, ZBTB22, SSBP3, TBX15, SOX13, ARID5B, ADNP, SPEN, ZNF689, UBN1, MCM6, HOXC10, BHMG1, LCOR, EEF1D, TBX18, TOP3B, ZNF276, SMARCAD1, IRX3, BCLAF1, ZFP64, THAP4, MGMT, GLIS1, NR3C1, ZBTB17, PRDM16, ZNF652, APLP2, HIC1, PRDM13, ZFP36L1, NPAS1, STAT4, REL, ZNF709, POU2F2, THAP1, HBP1, BCL6, MLXIP, GFI1, NFATC2, BAZ2B, FOXD1, SIM1, NFATC1, LMX1B, MSH4, PPP1R10, VSX2, ZNF358, TP73, DOT1L, ATXN1, NME1-NME2, MEOX2, ZNF362, CHTF18, NHLH2, IRF2, SP6, PHF21A, ZBTB3, NFIC | 1.117773 | 1 | 0.394416 |
| GOTERM_BP_DIRECT | GO:0006351~transcription, DNA-templated | 170 | 10.904 | 0.097802 | MMS19, ZNF581, ZNF584, CNOT8, CCDC85B, ZNF580, ASCC1, ZNF827, CBX3, NR2E3, MXI1, MIER2, ZFP90, MED26, ZNF397, RARB, ATOH8, SS18L1, CIITA, PRAME, ZNF790, MTA1, HMG20A, ARID1B, TBR1, FOXN3, KDM2A, HNF4A, PIAS4, ZNF783, PARP14, ZZZ3, TGIF1, ZNF382, ZNF132, TRRAP, XAB2, ZNF846, CXXC1, NR1H2, FOXQ1, AHRR, ZNF225, ZNF429, CASZ1, LHX5, NR1H3, ZNF529, EPAS1, ZNF621, AKAP8L, NR4A2, CIDEA, SKI, SMYD2, ZBTB45, MED13L, ZNF585A, SFMBT1, ZBTB44, ZBTB43, FOXP1, SREBF2, ZNF526, HDAC4, DMRTC2, HOXB7, DMTF1, SMARCC2, DNMT1, TCF19, HDAC9, ZFHX2, RERE, NCOR2, ZNF536, ELF2, FOSL2, FOXK1, WWC1, ZBTB37, BZW1, PCGF3, AES, HEY1, ZCCHC9, ZNF407, FOXB2, HSF4, NR2F2, LOXL2, ZBTB22, SSBP3, TBX15, POLR1E, SOX13, ARID5B, ADNP, TLE4, IRF2BP2, SPEN, POLR1B, IRF2BP1, ZNF689, DAPK3, BHMG1, HOXC10, NAB2, ZMIZ1, BRDT, VOPP1, USP22, LCOR, EEF1D, TBX18, ZNF276, ABLIM2, IRX3, BCLAF1, SBNO2, ZFP64, CTNND2, NR3C1, ZBTB17, PRDM16, SEC14L2, ZNF652, HIC1, PRDM13, NPAS1, STAT4, ZNF709, TSC22D4, POU2F2, GATAD2B, BCL3, THAP1, CC2D1B, MYCBP, HBP1, BCL6, MLXIP, GFI1, BCAS3, BAZ2B, SIM1, HSPA8, BAHD1, COL4A2, LMX1B, MAK, PPP1R10, PHF12, PARK2, VSX2, BIRC2, ZNF358, TP73, ATXN1, NME1-NME2, ZNF362, KDM4B, NHLH2, SP6, PHF21A, ZBTB3, KDM4A, NFIC, APBB1, ALKBH4 | 1.102018 | 1 | 0.990573 |
| GOTERM_MF_DIRECT | GO:0003677~DNA binding | 140 | 8.980 | 0.356522 | ZNF581, CDX1, ZNF580, ZNF827, MXI1, MIER2, ZFP90, MED26, RABGEF1, TIGD5, RARB, DDX20, FBXO21, CIITA, RBFOX3, POLE, ZNF790, HMG20A, ARID1B, TAF6L, TBR1, SIX6, RFC3, KDM2A, HNF4A, PIAS4, PRDM4, ZNF783, ZZZ3, TGIF1, LENEP, CAMTA2, ZNF132, AGFG1, MEIS1, CXXC1, ZNF846, NR1H2, AHRR, ZNF225, ZNF429, CERS2, CASZ1, STRBP, NTHL1, NR1H3, SREBF1, DHX9, ZNF529, TAF4, EPAS1, SPHK1, ZNF621, NR4A2, AKAP8L, TEAD1, ARID3B, TET2, ZBTB45, ZBTB44, ZBTB43, ZNF526, QRICH1, DNA2, HOXB7, DMTF1, SMARCC2, DNMT1, RERE, DNAJB6, NCOR2, IER2, KIF22, ARID4A, ZBTB37, HEY1, RTF1, ZNF407, NR2F2, KDM5A, IMPDH2, ZBTB22, ZFP36, POLR1E, KIAA1841, ARID5B, ADNP, CDK9, SPEN, POLR1B, ZNF689, UBN1, MCM6, TAF11, BHMG1, ASH1L, LCOR, EEF1D, TBX18, TOP3B, ZNF276, SMARCAD1, BCLAF1, ZFP64, THAP4, MGMT, NR3C1, ZBTB17, APLP2, ZNF652, PRDM13, ZFP36L1, NPAS1, STAT4, ZNF709, POU2F2, BCL3, HBP1, MLXIP, BAZ2B, NFATC2, TINF2, FOXD1, SIM1, NFATC1, MSH4, PPP1R10, TRIM26, RCAN1, VSX2, ZNF358, DOT1L, ATXN1, NME1-NME2, ZNF362, CHTF18, IRF2, SP6, ZBTB3, NFIC | 1.041145 | 1 | 0.990554 |
| GOTERM_BP_DIRECT | GO:0006355~regulation of transcription, DNA-templated | 119 | 7.633 | 0.538812 | ZNF581, CDX1, ZNF584, CNOT8, TMEM189-UBE2V1, ZNF580, SNIP1, ZNF827, ASCC1, MXI1, GTF2E1, MIER2, ZFP90, ZNF397, RARB, CIITA, ZNF790, MTA1, HMG20A, PPARGC1B, ELL2, KDM2A, PRDM4, PARP14, ZZZ3, TGIF1, ERC1, ZNF382, ZNF132, UBE2V1, TRRAP, MEIS1, WT1, CXXC1, ZNF846, AHRR, ZNF225, ZNF429, CASZ1, NR1H3, ZNF529, ZNF621, CREBBP, CIDEA, CELSR2, ZBTB45, ZNF585A, SFMBT1, ZBTB44, ZBTB43, SFMBT2, ZNF526, NOTCH2, HOXB7, DMRTC2, DMTF1, ZFHX2, ZFHX3, TAF1C, RSF1, ZBTB37, BZW1, PCGF3, HEY1, ZNF407, HSF4, ZBTB22, BRF1, SOX13, ADNP, IRF2BP2, IRF2BP1, ZNF689, DAPK3, HOXC10, TAF11, BHMG1, VOPP1, LCOR, EEF1D, ZNF276, IRX3, SBNO2, ZFP64, CTNND2, NR3C1, PRDM16, ZNF652, HIC1, ZFP36L1, STAT4, ZNF709, REXO4, POU2F2, MYCBP, HBP1, THAP1, GFI1, BAZ2B, NFATC2, KAT2A, LZTR1, LMX1B, MAK, RCAN1, VSX2, BIRC2, ZNF358, RPS6KA4, MEOX2, NME1-NME2, ZNF362, KDM4B, SP6, PHF21A, IRF2, ZBTB3, APBB1, ALKBH4 | 1.002734 | 1 | 0.999971 |

Table contains cluster enrichment score, category, enrichment term, the number and proportion of genes associated with the term, P for that term, the genes in the enrichment term, fold enrichment, the Bonferroni-adjusted P and the Benjamini-Hochberf FDR. Annotation clusters with FDR < 0.05 for at least one term were reported. GAD_DISEASE = The Genetic Association Database; UP_KEYWORDS = UniProtKB keywords; UP_SEQ_FEATURE = Sequence annotation (Features) – UniProt; GOTERM_MF_DIRECT = New Gene Ontolog category (GO Direct) molecular function; GOTERM_BP_DIRECT = New Gene Ontolog category (GO Direct) biological processes; INTERPRO = InterPro: the integrative protein signature database; SMART = Simple Modular Architecture Research Tool.

**Supplementary file 2D. Associations of 25 significant CpGs with risk of coronary heart disease among 880 participants without usage of blood pressure lowering drug.**

| Chr | Position | CpG | Gene | Relation to Gene | All participants | | |  | | Participants without drugs* | | |
| --- | --- | --- | --- | --- | --- | --- | --- | --- | --- | --- | --- | --- |
|  |  |  |  |  | Effect | P |  | | Effect | | P |  |
| 9 | 115513036 | cg23398826 | SNX30 | TSS200 | -0.003 | 1.57E-08 |  | | -0.004 | | 2.25E-08 |  |
| 3 | 49068057 | cg02386575 | IMPDH2 | TSS1500 | 0.006 | 9.61E-08 |  | | 0.005 | | 2.06E-05 |  |
|  |  |  | QRICH1 | Body |  |  |  | |  |  |  |  |
| 19 | 37329330 | cg10400937 | ZNF790 | TSS200 | 0.002 | 1.09E-05 |  | | 0.002 | | 1.72E-05 |  |
| 12 | 131758671 | cg20562821 | (RPS6P20**) | | 0.005 | 2.42E-05 |  | | 0.005 | | 1.43E-04 |  |
| 6 | 34855635 | cg08106661 | TAF11 | 1stExon | 0.003 | 3.16E-05 |  | | 0.003 | | 7.80E-05 |  |
|  |  |  | ANKS1A | TSS1500 |  |  |  | |  |  |  |  |
| 1 | 153203211 | cg11630610 | (MIR584**) | | 0.005 | 3.83E-05 |  | | 0.005 | | 8.43E-06 |  |
| 1 | 8426319 | cg20302171 | RERE | 5'UTR | -0.004 | 4.29E-05 |  | | -0.004 | | 1.24E-04 |  |
| 11 | 63909324 | cg26334131 | MACROD1 | Body | -0.005 | 4.44E-05 |  | | -0.006 | | 1.50E-05 |  |
| 20 | 2444631 | cg07560408 | SNORD119 | TSS1500 | -0.005 | 4.46E-05 |  | | -0.005 | | 5.84E-05 |  |
|  |  |  | SNRPB | Body |  |  |  | |  |  |  |  |
| 19 | 46522185 | cg21210537 | MIR769 | TSS200 | 0.004 | 4.85E-05 |  | | 0.005 | | 2.75E-05 |  |
| 20 | 60546782 | cg15833447 | (TAF4**) | | 0.006 | 5.55E-05 |  | | 0.006 | | 4.33E-05 |  |
| 11 | 94963255 | cg02591826 | LOC100129203 | TSS200 | 0.002 | 5.70E-05 |  | | 0.002 | | 1.08E-04 |  |
| 7 | 100861083 | cg16639138 | ZNHIT1 | 5'UTR/1stExon | 0.002 | 6.46E-05 |  | | 0.002 | | 1.33E-04 |  |
|  |  |  | PLOD3 | TSS200 |  |  |  | |  |  |  |  |
| 6 | 27863042 | cg01545454 | (HIST1H2BO**) | | 0.002 | 7.29E-05 |  | | 0.002 | | 7.34E-05 |  |
| 1 | 203242409 | cg07219103 | (CHIT1**) | | 0.002 | 7.35E-05 |  | | 0.002 | | 7.99E-05 |  |
| 22 | 23994996 | cg05681643 | GUSBP11 | Body | 0.004 | 7.42E-05 |  | | 0.004 | | 5.97E-04 |  |
| 2 | 88991375 | cg06358566 | RPIA | 1stExon | -0.002 | 7.74E-05 |  | | -0.003 | | 9.05E-06 |  |
| 2 | 162273185 | cg19583211 | TBR1 | 1stExon | -0.003 | 7.97E-05 |  | | -0.003 | | 1.81E-03 |  |
| 20 | 3613189 | cg10643850 | ATRN | Body | 0.004 | 8.04E-05 |  | | 0.005 | | 2.07E-05 |  |
| 17 | 17460905 | cg13311494 | PEMT | Body | -0.005 | 8.50E-05 |  | | -0.005 | | 5.42E-05 |  |
| 1 | 179852195 | cg11754670 | TOR1AIP1 | Body | 0.001 | 8.84E-05 |  | | 0.001 | | 2.43E-04 |  |
| 15 | 74928935 | cg05740632 | EDC3 | Body | -0.004 | 9.07E-05 |  | | -0.004 | | 2.74E-05 |  |
| 11 | 1972510 | cg08484100 | MRPL23 | Body | -0.004 | 9.19E-05 |  | | -0.004 | | 1.71E-03 |  |
| 1 | 52822428 | cg24792179 | CC2D1B | Body | 0.004 | 9.87E-05 |  | | 0.004 | | 3.01E-04 |  |
| 7 | 68973036 | cg22794712 | (LOC100507468**) | | -0.006 | 1.10E-04 |  | | -0.005 | | 7.36E-04 |  |

* A total of 100 participants who reported usage of blood pressure lowering drugs at baseline were excluded.

** For inter-genic CpG sites, R package FDb.InfiniumMethylation.hg19 was used to locate the nearest annotated gene.

Effect size estimates were calculated based on normalized methylation values, denoting the methylation difference between cases and controls. CpG = cytosine-phosphoguanine site; Chr = chromosome; EWAS = epigenome-wide association; WGCNA = weighted gene co-methylation network analysis; FDR = false discovery rate; TSS200 = within 200 bp from transcription start site; TSS1500 = within 1500 bp from transcription start site; Body = the CpG is in gene body; 1stExon = the first exon; and UTR = untranslated region.

**Supplementary file 2E. Association between quartile methylation level of identified CpGs and systolic blood pressure* (mmHg).**

|  | Quartile 2 | | Quartile 3 | | Quartile 4 | | P for trend |  | Mediation effect | |
| --- | --- | --- | --- | --- | --- | --- | --- | --- | --- | --- |
| CpGs | Effect size (se) | P | Effect size | P | Effect size | P |  |  | Proportion mediated, % | P |
| cg23398826 | -1.302 (2.078) | 0.531 | -4.176 (2.079) | 0.045 | -4.925 (2.076) | 0.018 | 0.004 |  | 5.88 | 0.016 |
| cg13311494 | -0.649 (2.051) | 0.752 | -0.301 (2.045) | 0.883 | -5.893 (2.087) | 0.005 | 0.063 |  | 11.19 | 0.081 |
| cg16639138 | 3.002 (2.053) | 0.144 | 3.277 (2.058) | 0.112 | 3.508 (2.050) | 0.087 | 0.132 |  | - | - |
| cg15833447 | 1.230 (2.058) | 0.550 | 1.197 (2.113) | 0.571 | 1.072 (2.109) | 0.612 | 0.143 |  | - | - |
| cg19583211 | 1.252 (2.081) | 0.547 | 3.777 (2.072) | 0.069 | 3.141 (2.080) | 0.131 | 0.182 |  | - | - |
| cg10643850 | -0.448 (2.079) | 0.829 | -2.578 (2.079) | 0.215 | -1.107 (2.128) | 0.603 | 0.184 |  | - | - |
| cg21210537 | 0.563 (2.106) | 0.789 | 2.170 (2.189) | 0.322 | 3.319 (2.249) | 0.140 | 0.292 |  | - | - |
| cg26334131 | 0.128 (2.082) | 0.951 | 2.211 (2.074) | 0.287 | 0.998 (2.117) | 0.637 | 0.297 |  | - | - |
| cg08106661 | 0.755 (2.138) | 0.724 | 1.032 (2.262) | 0.648 | -2.121 (2.416) | 0.380 | 0.299 |  | - | - |
| cg05681643 | -0.870 (2.045) | 0.671 | 2.518 (2.069) | 0.224 | 2.281 (2.142) | 0.287 | 0.335 |  | - | - |
| cg06358566 | -2.716 (2.066) | 0.189 | -0.781 (2.085) | 0.708 | -1.243 (2.079) | 0.550 | 0.365 |  | - | - |
| cg20562821 | -2.728 (2.094) | 0.193 | 0.636 (2.152) | 0.767 | -2.460 (2.179) | 0.259 | 0.501 |  | - | - |
| cg08484100 | 0.616 (2.056) | 0.765 | 0.386 (2.081) | 0.853 | 3.617 (2.097) | 0.085 | 0.504 |  | - | - |
| cg20302171 | -2.136 (2.063) | 0.301 | -0.761 (2.058) | 0.712 | 0.323 (2.099) | 0.878 | 0.525 |  | - | - |
| cg10400937 | 2.011 (2.059) | 0.329 | 2.397 (2.074) | 0.248 | 2.063 (2.103) | 0.327 | 0.543 |  | - | - |
| cg02386575 | 0.227 (2.028) | 0.911 | 1.498 (2.067) | 0.469 | 0.636 (2.073) | 0.759 | 0.551 |  | - | - |
| cg24792179 | -0.250 (2.066) | 0.904 | -0.076 (2.103) | 0.971 | 2.523 (2.150) | 0.241 | 0.604 |  | - | - |
| cg22794712 | -0.261 (2.074) | 0.900 | 0.996 (2.135) | 0.641 | -1.122 (2.167) | 0.605 | 0.608 |  | - | - |
| cg07219103 | 3.365 (2.071) | 0.105 | 1.553 (2.111) | 0.462 | 1.994 (2.202) | 0.365 | 0.658 |  | - | - |
| cg11754670 | 1.364 (2.087) | 0.513 | 0.706 (2.134) | 0.741 | -0.319 (2.154) | 0.882 | 0.673 |  | - | - |
| cg05740632 | -0.772 (2.060) | 0.708 | 1.094 (2.116) | 0.605 | 1.147 (2.125) | 0.589 | 0.779 |  | - | - |
| cg02591826 | -1.849 (2.059) | 0.369 | -0.045 (2.077) | 0.983 | -0.937 (2.124) | 0.659 | 0.854 |  | - | - |
| cg01545454 | -4.476 (2.055) | 0.030 | -2.310 (2.090) | 0.269 | -1.302 (2.061) | 0.528 | 0.873 |  | - | - |
| cg07560408 | -3.080 (2.080) | 0.139 | -2.120 (2.062) | 0.304 | -0.563 (2.082) | 0.787 | 0.886 |  | - | - |
| cg11630610 | 1.344 (2.086) | 0.520 | 0.206 (2.065) | 0.921 | 1.438 (2.081) | 0.490 | 0.981 |  | - | - |

*****Participants (n=100) who reported usage of blood pressure lowering medications were excluded.

CpG = cytosine-phosphoguanine site; SE = standard error. Effect size is for the comparison with quartile 1. Multivariable model was adjusted for: age, sex, education level, marital status, smoking, drinking, physical activity, diet score, body mass index, fasting time, study area, and batch. Mediation analysis was performed for significant CpGs only.

**Supplementary file 2F. Association between quartile methylation level of identified CpGs and diastolic blood pressure* (mmHg).**

|  | Quartile 2 | | Quartile 3 | | Quartile 4 | | P for trend |  | Mediation effect | |
| --- | --- | --- | --- | --- | --- | --- | --- | --- | --- | --- |
| CpGs | Effect size (se) | P | Effect size | P | Effect size | P |  |  | Proportion mediated, % | P |
| cg23398826 | -0.586 (1.203) | 0.626 | -1.949 (1.204) | 0.106 | -2.526 (1.202) | 0.036 | 0.007 |  | 4.51 | 0.028 |
| cg16639138 | 2.408 (1.184) | 0.042 | 3.002 (1.187) | 0.012 | 2.821 (1.183) | 0.017 | 0.045 |  | 12.49 | 0.068 |
| cg13311494 | -0.788 (1.188) | 0.507 | -1.726 (1.184) | 0.145 | -3.39 (1.208) | 0.005 | 0.046 |  | 9.87 | 0.070 |
| cg19583211 | -0.097 (1.202) | 0.936 | 2.369 (1.197) | 0.048 | 1.711 (1.201) | 0.155 | 0.153 |  | - | - |
| cg11630610 | 1.027 (1.204) | 0.394 | -1.275 (1.192) | 0.285 | -0.414 (1.201) | 0.730 | 0.274 |  | - | - |
| cg15833447 | -0.946 (1.190) | 0.426 | -0.301 (1.222) | 0.805 | 0.390 (1.219) | 0.749 | 0.289 |  | - | - |
| cg08106661 | -0.229 (1.237) | 0.853 | 0.568 (1.308) | 0.664 | -1.280 (1.397) | 0.360 | 0.365 |  | - | - |
| cg26334131 | 0.674 (1.205) | 0.576 | 0.933 (1.200) | 0.437 | 1.110 (1.224) | 0.365 | 0.382 |  | - | - |
| cg07219103 | 1.310 (1.199) | 0.275 | 0.969 (1.222) | 0.428 | 1.191 (1.275) | 0.350 | 0.401 |  | - | - |
| cg21210537 | 0.250 (1.219) | 0.838 | 0.835 (1.267) | 0.510 | 1.184 (1.302) | 0.363 | 0.432 |  | - | - |
| cg05681643 | 0.144 (1.184) | 0.903 | 0.288 (1.197) | 0.810 | 1.635 (1.240) | 0.187 | 0.457 |  | - | - |
| cg24792179 | -0.089 (1.195) | 0.941 | 0.239 (1.217) | 0.845 | 1.169 (1.244) | 0.348 | 0.509 |  | - | - |
| cg10643850 | 0.310 (1.203) | 0.796 | 0.316 (1.203) | 0.793 | -0.345 (1.232) | 0.779 | 0.524 |  | - | - |
| cg08484100 | 0.206 (1.190) | 0.863 | 0.238 (1.204) | 0.843 | 1.773 (1.214) | 0.144 | 0.540 |  | - | - |
| cg05740632 | 0.203 (1.191) | 0.865 | -0.179 (1.223) | 0.884 | 1.233 (1.229) | 0.316 | 0.562 |  | - | - |
| cg02591826 | -3.075 (1.183) | 0.009 | 0.751 (1.193) | 0.530 | -1.259 (1.221) | 0.303 | 0.657 |  | - | - |
| cg10400937 | 1.552 (1.190) | 0.192 | 0.905 (1.198) | 0.450 | 1.888 (1.215) | 0.121 | 0.657 |  | - | - |
| cg07560408 | -1.107 (1.204) | 0.358 | -0.766 (1.194) | 0.521 | -0.908 (1.206) | 0.452 | 0.689 |  | - | - |
| cg22794712 | -0.749 (1.198) | 0.532 | 0.227 (1.233) | 0.854 | -1.654 (1.252) | 0.187 | 0.694 |  | - | - |
| cg20562821 | -1.448 (1.212) | 0.232 | 0.442 (1.245) | 0.723 | -0.257 (1.261) | 0.838 | 0.726 |  | - | - |
| cg11754670 | 1.337 (1.206) | 0.268 | 0.696 (1.234) | 0.573 | 0.599 (1.245) | 0.630 | 0.757 |  | - | - |
| cg01545454 | -0.479 (1.190) | 0.687 | -1.233 (1.211) | 0.309 | 0.596 (1.194) | 0.618 | 0.763 |  | - | - |
| cg06358566 | -0.912 (1.195) | 0.445 | -1.093 (1.205) | 0.365 | 0.510 (1.202) | 0.671 | 0.874 |  | - | - |
| cg20302171 | -0.919 (1.193) | 0.441 | 0.106 (1.191) | 0.929 | -0.899 (1.214) | 0.459 | 0.956 |  | - | - |
| cg02386575 | -0.575 (1.173) | 0.624 | -0.798 (1.195) | 0.505 | 0.007 (1.199) | 0.996 | 0.995 |  | - | - |

*****Participants (n=100) who reported usage of blood pressure lowering medications were excluded.

CpG = cytosine-phosphoguanine site; SE = standard error. Effect size is for the comparison with quartile 1. Multivariable model was adjusted for: age, sex, education level, marital status, smoking, drinking, physical activity, diet score, body mass index, fasting time, study area, and batch. Mediation analysis was performed for significant CpGs only.

**Supplementary file 2G. The annotated or nearest annotated gene of the identified CHD-associated CpGs in our study and the previous GWAS findings**

| Gene | CpG | Associations reported in GWAS catalog | | | | |
| --- | --- | --- | --- | --- | --- | --- |
|  |  | Cardiovascular disease | Cardiometabolic traits | Physical measurement | Lifestyle | Others established or potential risk factor of coronary heart disease |
| SNX30 | cg23398826 |  | Diastolic blood pressure night-to-day ratio in hypertension^1^; Fasting plasma glucose^2^ |  |  |  |
| IMPDH2 | cg02386575 |  |  |  |  | Educational attainment^3^ |
| QRICH1 |  | Coronary heart disease^4^ |  |  | s | Macrophage inflammatory protein 1b^5^; Mood instability^6^ |
| ZNF790 | cg10400937 |  |  |  |  |  |
| (RPS6P20*) | cg20562821 |  |  |  |  |  |
| TAF11 | cg08106661 |  | Fasting blood insulin^7^ | Height^8^ |  |  |
| ANKS1A |  | Coronary heart disease^9,10^ |  | Height^11^; Body mass index^8^; Waist circumference adjusted for body mass index^12,13^ | Alcoholism^14^; Smoking cessation^15^ | Bone mineral density^16^ |
| (MIR584*) | cg11630610 |  |  |  |  |  |
| RERE | cg20302171 | Cardiovascular disease^8^ | Systolic blood pressure^8^ | Height^8^; Body fat percentage^17^ | Smoking initiation^8,18^ | Educational attainment^19,20^; Bone mineral density^21,22^; Depression^23,24^; Asthma^25,26^; Life satisfaction^27,28^; Menarche (age at onset)^8^ |
| MACROD1 | cg26334131 | Cardiovascular disease^8^ | Triglyceride^29,30^; High density lipoprotein cholesterol^29,30^ | Waist-to-hip ratio adjusted for body mass index^12^; Body mass index^31^ | Lipid level x physical activity interaction^32^; Lipid level x alcohol interaction^33^; | General cognitive ability^3,34^; Serum uric acid levels^35^ |
| SNORD119 | cg07560408 |  |  |  |  |  |
| SNRPB |  |  |  |  |  |  |
| MIR769 | cg21210537 |  |  |  |  |  |
| (TAF4*) | cg15833447 |  | Systolic blood pressure^8^ | Body mass index^36^; Height^8^ |  | Plasma factor VII activating protease levels^37^ |
| LOC100129203 | cg02591826 |  |  |  |  |  |
| ZNHIT1 | cg16639138 |  |  |  |  | Proteome^38^ |
| PLOD3 |  |  |  |  |  |  |
| (HIST1H2BO*) | cg01545454 |  |  |  |  |  |
| (CHIT1*) | cg07219103 |  |  | Hip circumference adjusted for body mass index^39^ |  | Proteome^38,40^ |
| GUSBP11 | cg05681643 |  |  |  |  |  |
| RPIA | cg06358566 |  |  | Height^39^ |  |  |
| TBR1 | cg19583211 | ; | Systolic blood pressure^8,41^ |  |  | Educational attainment^3^ |
| ATRN | cg10643850 |  |  |  |  |  |
| PEMT | cg13311494 |  | Triglycerides^42^ | Waist-hip ratio^12,43^; Height^44^ |  |  |
| TOR1AIP1 | cg11754670 |  |  |  |  |  |
| EDC3 | cg05740632 |  |  |  |  |  |
| MRPL23 | cg08484100 |  | Systolic blood pressure x alcohol consumption interaction^45^ |  |  |  |
| CC2D1B | cg24792179 |  |  |  |  |  |
| (LOC100507468*) | cg22794712 |  |  |  |  |  |
| PLXNB2 |  |  | Systolic blood pressure^41^ | Body mass index^8^ |  | Proteome^40^ |

* For inter-genic CpG sites, R package FDb.InfiniumMethylation.hg19 was used to locate the nearest annotated gene.

**Supplemental** References

1. Rimpelä JM, Pörsti IH, Jula A, et al. Genome-wide association study of nocturnal blood pressure dipping in hypertensive patients. BMC Med Genet 2018;19(1):110.

2. Ge S, Wang Y, Song M, et al. Type 2 Diabetes Mellitus: Integrative Analysis of Multiomics Data for Biomarker Discovery. Omics J Integr Biol 2018;22(7):514–23.

3. Lee JJ, Wedow R, Okbay A, et al. Gene discovery and polygenic prediction from a genome-wide association study of educational attainment in 1.1 million individuals. Nat Genet 2018;50(8):1112–21.

4. van der Harst P, Verweij N. Identification of 64 Novel Genetic Loci Provides an Expanded View on the Genetic Architecture of Coronary Artery Disease. Circ Res 2018;122(3):433–43.

5. Ahola-Olli AV, Würtz P, Havulinna AS, et al. Genome-wide Association Study Identifies 27 Loci Influencing Concentrations of Circulating Cytokines and Growth Factors. Am J Hum Genet 2017;100(1):40–50.

6. Ward J, Tunbridge EM, Sandor C, et al. The genomic basis of mood instability: identification of 46 loci in 363,705 UK Biobank participants, genetic correlation with psychiatric disorders, and association with gene expression and function. Mol Psychiatry 2019;

7. Manning AK, Hivert M-F, Scott RA, et al. A genome-wide approach accounting for body mass index identifies genetic variants influencing fasting glycemic traits and insulin resistance. Nat Genet 2012;44(6):659–69.

8. Kichaev G, Bhatia G, Loh P-R, et al. Leveraging Polygenic Functional Enrichment to Improve GWAS Power. Am J Hum Genet 2019;104(1):65–75.

9. Dichgans M, Malik R, König IR, et al. Shared genetic susceptibility to ischemic stroke and coronary artery disease: a genome-wide analysis of common variants. Stroke 2014;45(1):24–36.

10. Schunkert H, König IR, Kathiresan S, et al. Large-scale association analysis identifies 13 new susceptibility loci for coronary artery disease. Nat Genet 2011;43(4):333–8.

11. Berndt SI, Gustafsson S, Mägi R, et al. Genome-wide meta-analysis identifies 11 new loci for anthropometric traits and provides insights into genetic architecture. Nat Genet 2013;45(5):501–12.

12. Justice AE, Winkler TW, Feitosa MF, et al. Genome-wide meta-analysis of 241,258 adults accounting for smoking behaviour identifies novel loci for obesity traits. Nat Commun 2017;8:14977.

13. Shungin D, Winkler TW, Croteau-Chonka DC, et al. New genetic loci link adipose and insulin biology to body fat distribution. Nature 2015;518(7538):187–96.

14. Heath AC, Whitfield JB, Martin NG, et al. A quantitative-trait genome-wide association study of alcoholism risk in the community: findings and implications. Biol Psychiatry 2011;70(6):513–8.

15. Liu M, Jiang Y, Wedow R, et al. Association studies of up to 1.2 million individuals yield new insights into the genetic etiology of tobacco and alcohol use. Nat Genet 2019;51(2):237–44.

16. Pei Y-F, Liu L, Liu T-L, et al. Joint Association Analysis Identified 18 New Loci for Bone Mineral Density. J Bone Miner Res Off J Am Soc Bone Miner Res 2019;34(6):1086–94.

17. Hübel C, Gaspar HA, Coleman JRI, et al. Genomics of body fat percentage may contribute to sex bias in anorexia nervosa. Am J Med Genet Part B Neuropsychiatr Genet Off Publ Int Soc Psychiatr Genet 2019;180(6):428–38.

18. Erzurumluoglu AM, Liu M, Jackson VE, et al. Meta-analysis of up to 622,409 individuals identifies 40 novel smoking behaviour associated genetic loci. Mol Psychiatry 2019;

19. Rietveld CA, Esko T, Davies G, et al. Common genetic variants associated with cognitive performance identified using the proxy-phenotype method. Proc Natl Acad Sci U S A 2014;111(38):13790–4.

20. Okbay A, Beauchamp JP, Fontana MA, et al. Genome-wide association study identifies 74 loci associated with educational attainment. Nature 2016;533(7604):539–42.

21. Kemp JP, Morris JA, Medina-Gomez C, et al. Identification of 153 new loci associated with heel bone mineral density and functional involvement of GPC6 in osteoporosis. Nat Genet 2017;49(10):1468–75.

22. Medina-Gomez C, Kemp JP, Trajanoska K, et al. Life-Course Genome-wide Association Study Meta-analysis of Total Body BMD and Assessment of Age-Specific Effects. Am J Hum Genet 2018;102(1):88–102.

23. Nagel M, Jansen PR, Stringer S, et al. Meta-analysis of genome-wide association studies for neuroticism in 449,484 individuals identifies novel genetic loci and pathways. Nat Genet 2018;50(7):920–7.

24. Wray NR, Ripke S, Mattheisen M, et al. Genome-wide association analyses identify 44 risk variants and refine the genetic architecture of major depression. Nat Genet 2018;50(5):668–81.

25. Shrine N, Portelli MA, John C, et al. Moderate-to-severe asthma in individuals of European ancestry: a genome-wide association study. Lancet Respir Med 2019;7(1):20–34.

26. Ferreira MAR, Mathur R, Vonk JM, et al. Genetic Architectures of Childhood- and Adult-Onset Asthma Are Partly Distinct. Am J Hum Genet 2019;104(4):665–84.

27. Okbay A, Baselmans BML, De Neve J-E, et al. Genetic variants associated with subjective well-being, depressive symptoms, and neuroticism identified through genome-wide analyses. Nat Genet 2016;48(6):624–33.

28. Baselmans BML, Jansen R, Ip HF, et al. Multivariate genome-wide analyses of the well-being spectrum. Nat Genet 2019;51(3):445–51.

29. Qi G, Chatterjee N. Heritability informed power optimization (HIPO) leads to enhanced detection of genetic associations across multiple traits. PLoS Genet 2018;14(10):e1007549.

30. Hoffmann TJ, Theusch E, Haldar T, et al. A large electronic-health-record-based genome-wide study of serum lipids. Nat Genet 2018;50(3):401–13.

31. Locke AE, Kahali B, Berndt SI, et al. Genetic studies of body mass index yield new insights for obesity biology. Nature 2015;518(7538):197–206.

32. Kilpeläinen TO, Bentley AR, Noordam R, et al. Multi-ancestry study of blood lipid levels identifies four loci interacting with physical activity. Nat Commun 2019;10(1):376.

33. de Vries PS, Brown MR, Bentley AR, et al. Multiancestry Genome-Wide Association Study of Lipid Levels Incorporating Gene-Alcohol Interactions. Am J Epidemiol 2019;188(6):1033–54.

34. Davies G, Lam M, Harris SE, et al. Study of 300,486 individuals identifies 148 independent genetic loci influencing general cognitive function. Nat Commun 2018;9(1):2098.

35. Kanai M, Akiyama M, Takahashi A, et al. Genetic analysis of quantitative traits in the Japanese population links cell types to complex human diseases. Nat Genet 2018;50(3):390–400.

36. Akiyama M, Okada Y, Kanai M, et al. Genome-wide association study identifies 112 new loci for body mass index in the Japanese population. Nat Genet 2017;49(10):1458–67.

37. Olsson M, Stanne TM, Pedersen A, et al. Genome-wide analysis of genetic determinants of circulating factor VII-activating protease (FSAP) activity. J Thromb Haemost JTH 2018;16(10):2024–34.

38. Emilsson V, Ilkov M, Lamb JR, et al. Co-regulatory networks of human serum proteins link genetics to disease. Science 2018;361(6404):769–73.

39. Tachmazidou I, Süveges D, Min JL, et al. Whole-Genome Sequencing Coupled to Imputation Discovers Genetic Signals for Anthropometric Traits. Am J Hum Genet 2017;100(6):865–84.

40. Sun BB, Maranville JC, Peters JE, et al. Genomic atlas of the human plasma proteome. Nature 2018;558(7708):73–9.

41. Evangelou E, Warren HR, Mosen-Ansorena D, et al. Genetic analysis of over 1 million people identifies 535 new loci associated with blood pressure traits. Nat Genet 2018;50(10):1412–25.

42. Klarin D, Damrauer SM, Cho K, et al. Genetics of blood lipids among ~300,000 multi-ethnic participants of the Million Veteran Program. Nat Genet 2018;50(11):1514–23.

43. Justice AE, Karaderi T, Highland HM, et al. Protein-coding variants implicate novel genes related to lipid homeostasis contributing to body-fat distribution. Nat Genet 2019;51(3):452–69.

44. Rüeger S, McDaid A, Kutalik Z. Evaluation and application of summary statistic imputation to discover new height-associated loci. PLoS Genet 2018;14(5):e1007371.

45. Feitosa MF, Kraja AT, Chasman DI, et al. Novel genetic associations for blood pressure identified via gene-alcohol interaction in up to 570K individuals across multiple ancestries. PloS One 2018;13(6):e0198166.
