# Supplementary material for: Epigenome-wide analysis of DNA methylation and coronary heart disease: a nested case-control study
Source: eLife. 2021 Sep 13;10:e68671. doi: 10.7554/eLife.68671 (PMC8585480; doi:10.7554/eLife.68671)
Supplement: Transparent reporting form [file elife-68671-transrepform1.docx]

***eLife’s* transparent reporting form**

We encourage authors to provide detailed information *within their submission* to facilitate the interpretation and replication of experiments. Authors can upload supporting documentation to indicate the use of appropriate reporting guidelines for health-related research (see [EQUATOR Network](http://www.equator-network.org/%20)), life science research (see the [BioSharing Information Resource](https://biosharing.org/)), or the [ARRIVE guidelines](http://www.plosbiology.org/article/info:doi/10.1371/journal.pbio.1000412) for reporting work involving animal research. Where applicable, authors should refer to any relevant reporting standards documents in this form.

If you have any questions, please consult our Journal Policies and/or contact us: [editorial@elifesciences.org](mailto:editorial@elifesciences.org).

**Sample-size estimation**

- You should state whether an appropriate sample size was computed when the study was being designed
- You should state the statistical method of sample size computation and any required assumptions
- If no explicit power analysis was used, you should describe how you decided what sample (replicate) size (number) to use

Please outline where this information can be found within the submission (e.g., sections or figure legends), or explain why this information doesn’t apply to your submission:

The present analysis is based on an established population-based cohort study, CKB. We included as many eligible individuals as possible and didn’t calculate the sample size. Information on inclusion and exclusion criteria can be found in the subsection of ‘Study population’ and ‘Study design’ under ‘Materials and Methods’ part.

**Replicates**

- You should report how often each experiment was performed
- You should include a definition of biological versus technical replication
- The data obtained should be provided and sufficient information should be provided to indicate the number of independent biological and/or technical replicates
- If you encountered any outliers, you should describe how these were handled
- Criteria for exclusion/inclusion of data should be clearly stated
- High-throughput sequence data should be uploaded before submission, with a private link for reviewers provided (these are available from both GEO and ArrayExpress)

Please outline where this information can be found within the submission (e.g., sections or figure legends), or explain why this information doesn’t apply to your submission:

This information can be found within the subsection of ‘Measurement of DNA methylation; Assessment of covariates’ under ‘Materials and Methods’ part.

**Statistical reporting**

- Statistical analysis methods should be described and justified
- Raw data should be presented in figures whenever informative to do so (typically when N per group is less than 10)
- For each experiment, you should identify the statistical tests used, exact values of N, definitions of center, methods of multiple test correction, and dispersion and precision measures (e.g., mean, median, SD, SEM, confidence intervals; and, for the major substantive results, a measure of effect size (e.g., Pearson's r, Cohen's d)
- Report exact p-values wherever possible alongside the summary statistics and 95% confidence intervals. These should be reported for all key questions and not only when the p-value is less than 0.05.

Please outline where this information can be found within the submission (e.g., sections or figure legends), or explain why this information doesn’t apply to your submission:

This information can be found within the section of ‘Materials and Methods – Statistical analysis’.

(For large datasets, or papers with a very large number of statistical tests, you may upload a single table file with tests, Ns, etc., with reference to sections in the manuscript.)

**Group allocation**

- Indicate how samples were allocated into experimental groups (in the case of clinical studies, please specify allocation to treatment method); if randomization was used, please also state if restricted randomization was applied
- Indicate if masking was used during group allocation, data collection and/or data analysis

Please outline where this information can be found within the submission (e.g., sections or figure legends), or explain why this information doesn’t apply to your submission:

This is an observational study, and allocation is not applicable.

**Additional data files (“source data”)**

- We encourage you to upload relevant additional data files, such as numerical data that are represented as a graph in a figure, or as a summary table
- Where provided, these should be in the most useful format, and they can be uploaded as “Source data” files linked to a main figure or table
- Include model definition files including the full list of parameters used
- Include code used for data analysis (e.g., R, MatLab)
- Avoid stating that data files are “available upon request”

Please indicate the figures or tables for which source data files have been provided:

According to the Regulation of the People's Republic of China on the Administration of Human Genetic Resources, we are not allowed to provide Chinese human clinical and genetic data abroad without an official approval.

According to our previous experience, the Administration of Human Genetic Resources of China suggests obtaining official approval after the acceptance of paper. We can make the raw data of part data (significant CpGs that were found in our study) not all data available. The process of obtaining official approval usually takes 2-3 months.

For researchers who are interested to access the original data, the access policy and procedures are available at www.ckbiobank.org. In brief, the China Kadoorie Biobank (CKB) is being conducted jointly by the Clinical Trial Service Unit (CTSU), Nuffield Department of Population Health, University of Oxford, and Chinese Academy of Medical Sciences (CAMS) in Beijing. Requesters should be employees of a recognised academic institution, health service organisation or charitable research organisation with experience in medical research. Requestors should be able to demonstrate, through their peer reviewed publications in the area of interest, their ability to carry out the proposed study. After registration, details of the required information are provided on the CKB Data Access System. The CKB Access Team will review and respond to data requests within 6-8 weeks.

We are providing our syntax of statistical analysis and the source data for Table 3 and Table 4. We can also upload summary statistics for all 747,726 CpG sites as source data for Table 2 if needed. Figure 2 is the direct output from R software. We can provide the output file if needed.
